# Comparative Impact Response, Damage, and Healing Performance of CFRP Laminates with Different Thicknesses and Reinforcement Architectures

**DOI:** 10.3390/polym18202532

**Published:** 2026-10-15

**Authors:** Maryna Shevtsova, Valentin Rougier, Véronique Michaud, Oleksii Vambol, Svitlana Purhina

**Affiliations:** 1 Laboratory of Composite Materials, Vilnius Gediminas Technical University, 10223 Vilnius, Lithuania; 2 Laboratory for Processing of Advanced Composites, École Polytechnique Fédérale de Lausanne, 1015 Lausanne, Switzerland; 3 Department of Aviation and Manufacturing Engineering, Lviv Polytechnic National University, 79013 Lviv, Ukraine; oleksii.o.vambol@lpnu.ua (O.V.);

**Keywords:** carbon fiber-reinforced polymer, low-velocity impact, impact damage, self-healing, compression-after-impact, residual strength, aerospace composites

## Abstract

Carbon-fiber-reinforced polymer (CFRP) laminates can exhibit different low-velocity impact responses and post-impact performance depending on laminate configuration. This study compares three self-healing CFRP configurations manufactured from CompPair HealTech^®^ materials: 1.45 and 3.30 mm unidirectional (UD) laminates and a 2.66 mm woven laminate. Low-velocity impact tests were performed at 5 and 10 J. Internal damage was qualitatively examined using visual inspection and X-ray phase-contrast imaging (XPCI), while post-impact performance and recovery were assessed by compression-after-impact (CAI) testing before and after thermal healing. The 1.45 mm UD laminate exhibited the largest displacement and lowest maximum impact force, whereas the 3.30 mm UD laminate showed the highest force and smallest displacement, with the woven laminate exhibiting an intermediate response. Despite its greater impact stiffness, the 3.30 mm UD laminate showed the largest relative reduction in residual compressive strength after 10 J, with strength retention decreasing from approximately 98% after 5 J to 64% after 10 J. The 1.45 mm UD and woven laminates retained 89% and 88% after 5 J and 82% and 72% after 10 J, respectively. After healing, the 1.45 mm UD and woven laminates recovered to approximately 99% of their virgin strengths after 5 J. After 10 J, strength retention reached 91%, 79%, and 81% for the 1.45 mm UD, 3.30 mm UD, and woven laminates, with corresponding healing efficiencies of 49%, 42%, and 31%, respectively. XPCI revealed configuration-dependent internal damage patterns that became more pronounced at 10 J. Overall, the investigated laminate configurations exhibited distinct impact responses, internal damage patterns, residual strengths, and levels of mechanical recovery after healing.

## 1. Introduction

Carbon-fiber-reinforced polymer (CFRP) laminates are widely used in aerospace structures because of their high specific stiffness and strength, corrosion resistance, and the ability to tailor mechanical properties through reinforcement architecture and stacking sequence. These characteristics are particularly advantageous for unmanned aerial vehicles (UAVs), where structural mass directly affects payload capacity, flight endurance, maneuverability, and energy consumption. Accordingly, thin CFRP laminates are commonly considered for aircraft and UAV skins, control surfaces, fairings, access panels, and other lightweight structural components. However, reducing laminate thickness to minimize structural mass can increase susceptibility to transverse impact loading and the development of barely visible impact damage (BVID) [1,2,3].

Low-velocity impacts can occur during manufacturing, assembly, maintenance, transportation, take-off and landing, or service, with typical sources including tool drops, runway debris, hail, handling damage, and foreign-object strikes. Although such events may produce only a small surface indentation, they can generate extensive subsurface damage, including matrix cracking, interfacial debonding, ply splitting, delamination, and, at higher impact severities, fiber fracture [1,2,4]. This internal damage is particularly detrimental under subsequent compression because delaminated sublaminates can buckle locally, leading to rapid damage propagation and a pronounced reduction in load-carrying capacity. Consequently, compression-after-impact (CAI) performance is widely used to assess the damage tolerance and residual strength of aerospace composite laminates [5,6,7].

For lightweight aerospace and UAV structures, the consequences of impact damage extend beyond the immediate reduction in load-carrying capability. Undetected subsurface damage may persist during subsequent service, while conventional CFRP laminates generally require inspection and, when damage exceeds allowable limits, repair or replacement. Self-healing composites offer an alternative approach by enabling partial recovery of mechanical performance after damage, potentially extending service life and reducing maintenance requirements. This makes the assessment of both residual performance and its subsequent recovery particularly relevant for the development of next-generation lightweight aerospace composites.

Self-healing strategies for fiber-reinforced polymer composites can generally be classified as intrinsic and extrinsic approaches. Extrinsic systems, including microcapsule- and vascular-based concepts, release or deliver healing agents to damaged regions, whereas intrinsic approaches exploit reversible interactions or thermally activated mobility of the polymer system [8,9]. Microcapsule-based systems can provide autonomous healing after crack-induced release of the healing agent, but the amount of healing agent available locally is finite. Vascular systems can supply healing agents to larger damaged regions and may allow repeated healing, although this requires the incorporation of a more complex delivery network into the composite [8,10]. Thermally activated thermoplastic-based systems offer another approach, in which a healing phase already incorporated into the matrix is mobilized by heating. In phase-separated epoxy–thermoplastic systems, heating above the melting range of the thermoplastic phase promotes its expansion, flow, and wetting of newly formed crack surfaces, allowing matrix cracks and delaminated regions to be partially refilled and bridged [11,12]. An important advantage of this approach is that the healing phase is already distributed within the material and can potentially be activated more than once, without introducing an external repair material. However, the effectiveness of thermal healing depends on the amount and mobility of the healing phase, crack opening, and damage severity. Recovery becomes limited when the available healing phase cannot completely fill the damaged region or when irreversible damage, particularly fiber fracture, contributes substantially to the loss of mechanical performance [12].

CompPair Technologies SA has developed a self-healing composite material system, in which the healing functionality is integrated into the composite material and activated by an appropriate thermal treatment. Such a system provides an opportunity to investigate the extent to which impact-induced degradation of CFRP laminates can be reversed. Because recovery may depend on the initial damage state, laminate configuration is an important consideration when evaluating healing performance. Quantitative healing metrics are therefore useful for distinguishing differences in recovery between composite configurations [13]. In particular, consideration of healing efficiency together with absolute residual strength and normalized strength retention can provide a more informative assessment of post-impact performance.

The response of a CFRP laminate to low-velocity impact results from the interaction between local damage mechanisms and global structural deformation. Parameters such as contact stiffness, peak impact force, maximum displacement, contact duration, energy absorption, and damage morphology depend on laminate thickness, bending stiffness, stacking sequence, fiber orientation, matrix properties, impact energy, impactor geometry, and boundary conditions [1,14,15]. Laminate thickness affects both the global deformation response and the distribution of stresses generated during impact. Relatively thick laminates generally exhibit greater bending and contact stiffness, resulting in higher impact-force levels and lower out-of-plane displacement, whereas thin laminates can undergo greater global deformation and exhibit a different balance between structural deformation and local damage formation [1,4,16,17,18]. Increasing thickness, therefore, does not necessarily translate into a proportional improvement in damage tolerance, as it also modifies interlaminar stress distributions, delamination development, and energy-dissipation mechanisms [1,2,16].

Reinforcement architecture provides an additional means of controlling impact-induced damage. Unidirectional (UD) plies offer considerable freedom for tailoring in-plane stiffness and strength through ply orientation, but their impact response can be strongly influenced by interlaminar stresses, ply splitting, and delamination at interfaces between differently oriented plies. Woven reinforcements, in contrast, introduce fiber interlacing and greater structural continuity between the principal in-plane directions. This architecture can influence crack propagation and local load redistribution, while fiber crimp, resin-rich regions, and weave geometry affect stiffness and damage initiation [19,20,21]. Consequently, UD and woven CFRP laminates may exhibit different combinations of impact response, damage extent, and residual compressive performance. These differences are particularly relevant when laminate thickness and reinforcement architecture vary simultaneously.

Although the effects of laminate thickness, stacking sequence, and reinforcement architecture on low-velocity impact behavior have been extensively investigated [1,2,20], direct comparisons between practically relevant CFRP configurations remain difficult, particularly when their impact response, internal damage, residual compressive performance, and post-impact recovery are considered together. Differences in thickness, cross-sectional area, and reinforcement architecture inherently affect absolute impact force and load-carrying capacity. Therefore, a higher absolute residual strength does not necessarily indicate better damage tolerance, while a thinner laminate may retain a greater proportion of its original strength despite exhibiting a lower absolute load-carrying capacity. Meaningful comparison should consequently consider both absolute and normalized mechanical performance together with the underlying damage state.

Mechanical impact parameters alone, however, cannot fully describe the severity or morphology of the resulting damage. Visual inspection is generally insufficient because the size of a surface indentation does not necessarily represent the extent of subsurface damage in CFRP laminates [22,23,24]. Advanced X-ray imaging is particularly useful for relating the impact response to the internal architecture and damage state of a composite. Grating-based dark-field imaging has been demonstrated to provide information on fiber-bundle orientation and laminate structure, including features associated with woven architectures that are difficult to resolve using conventional absorption imaging [25]. In impact-damaged CFRP, multimodal X-ray imaging combining absorption, differential phase, and dark-field signals has enabled the visualization of delaminations, fiber-direction damage, and smaller-scale defects, providing complementary information across different length scales [25,26,27]. Recent applications have further demonstrated the capability of advanced X-ray imaging to reveal damage mechanisms in CFRP laminates with different ply architectures [28]. These capabilities are particularly relevant to the present study because the investigated laminates differ not only in thickness but also in reinforcement architecture. XPCI therefore provides a means of examining whether differences in the measured impact response and subsequent CAI performance are accompanied by distinct internal damage patterns that cannot be identified reliably from surface inspection alone.

For self-healing CFRP laminates, the relationship between the initial damage state, residual mechanical performance, and subsequent recovery is particularly important. Combining mechanical impact measurements with direct observations of internal damage can therefore provide a more complete basis for interpreting post-impact performance and assessing the effectiveness of healing. Quantitative comparison using both absolute residual strength and normalized strength retention, together with healing efficiency, can help distinguish differences in the initial damage state from differences in the extent of mechanical recovery.

Accordingly, the present study provides a comparative assessment of three self-healing CFRP laminate configurations rather than a factorial investigation of the independent effects of thickness and reinforcement architecture. Because the investigated configurations differ simultaneously in laminate thickness, reinforcement architecture, and constituent material system, the observed differences are interpreted as configuration-dependent responses and are not attributed independently to any single parameter. The experimental results for the 1.45 mm UD laminate were previously reported in [29]. The new contribution of the present study consists of experimental results for the 3.30 mm UD and 2.66 mm woven laminates and a comparative assessment of all three configurations. The investigated configurations comprise 1.45 mm and 3.30 mm unidirectional laminates and a 2.66 mm woven laminate subjected to impact energies of 5 and 10 J. Their impact response was evaluated using force–time, displacement–time, energy–time, and force–displacement relationships, while the damage was characterized using optical inspection and multimodal X-ray phase-contrast imaging. Residual compressive performance was assessed by compression-after-impact testing before and after thermal healing. The study examines how differences in global impact response and internal damage are reflected in residual compressive strength and subsequent recovery, using both normalized strength retention and healing efficiency to compare the post-impact performance of the investigated configurations for aerospace and UAV applications.

## 2. Materials and Methods

**Figure 1:**
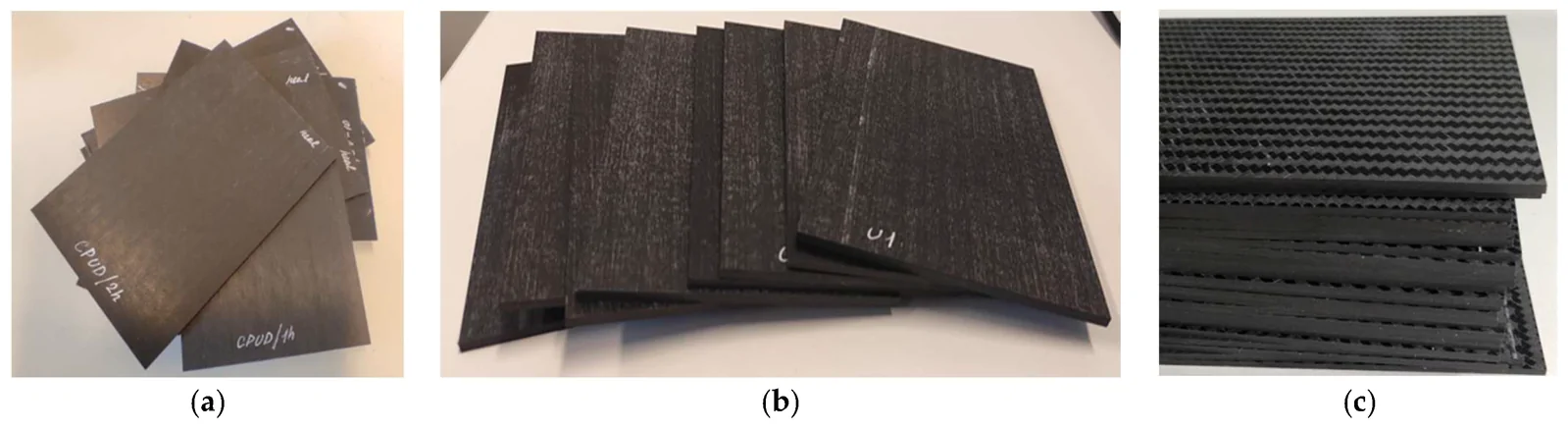
Specimens used for low-velocity impact and compression-after-impact testing: (**a**) CompPair UD laminate with a nominal thickness of 1.45 mm; (**b**) CompPair UD laminate with a nominal thickness of 3.30 mm; (**c**) CompPair woven laminate with a nominal thickness of 2.66 mm.

Three CFRP laminate configurations differing in thickness and reinforcement architecture were investigated (Figure 1). The two UD laminates used unidirectional carbon-fiber prepreg CompPair HealTech^®^ T700-UD200-36RW-300 (CompPair Technologies Ltd., Renens, Switzerland) with the same balanced, symmetric orientation pattern [0/+45/90/−45/−45/90/+45/0]. The 1.45 ± 0.02 mm UD laminate contained one such sequence, whereas the 3.30 ± 0.05 mm UD laminate contained twice as many plies arranged by repeating the same orientation pattern. A third laminate was manufactured from CompPair HealTech^®^ T300-TW200-42RW-1250 (CompPair Technologies Ltd., Renens, Switzerland) woven carbon-fiber prepreg, with ply orientation [+45/−45/0/90/+45/−45/0/90/+45/−45/0/90/+45/−45], and had a final thickness of 2.66 ± 0.05 mm. Although both UD laminates were manufactured from the same prepreg type, their average cured ply thicknesses differed (approximately 0.181 mm for UD-1.45 and 0.206 mm for UD-3.30). Different auxiliary materials were used during vacuum-bag processing, which may have affected laminate consolidation and the resulting thickness. However, the contribution of individual processing factors was not quantified. The fiber volume fractions and void contents of the manufactured laminates were not independently measured. Therefore, possible differences in these characteristics cannot be excluded as additional factors influencing the observed mechanical responses. Note that the fiber type was different between the UD (T700) and the woven (T300); however, these have the same modulus of 230 GPa, allowing for stiffness comparison, and different nominal strength (4.9 GPa versus 3.93 GPa). The CompPair HealTech^®^ material system incorporates a thermally activated healing functionality intended to promote recovery of matrix- and interface-related damage. However, the specific formulation and underlying chemical mechanisms of the proprietary HealTech^®^ technology have not been disclosed by the manufacturer. Therefore, the present study focuses on the experimentally observed recovery of mechanical performance rather than on identifying the molecular mechanisms responsible for healing. Table 1 summarizes the main material parameters of the samples, and Table 2 presents the calculated laminate properties. The laminate properties were determined using the classical laminate theory (CLT). The reduced stiffness matrix *Q* of an individual orthotropic ply was first calculated from the measured elastic properties *E*_1_, *E*_2_, *G*_12_, and *ν*_12_. For each ply, the stiffness matrix was then transformed according to its fiber orientation to obtain the corresponding transformed reduced stiffness matrix $\bar{Q}$. The laminate extensional, coupling and bending stiffness matrices (*A*, *B* and *D*, respectively) were subsequently calculated by integrating the transformed ply stiffnesses through the laminate thickness:

$$A\;\;=\;\;\sum_{k=1}^{n}{\bar{Q}}^{\left(k\right)}\;\;\left(z_{k}-z_{k-1}\right)$$

(1) $$B\;=\;\frac{1}{2}\;\;\sum_{k=1}^{n}{\bar{Q}}^{\left(k\right)}\;\;\left(z_{k}^{2}-z_{k-1}^{2}\right)$$

$$D\;=\;\frac{1}{3}\;\;\sum_{k=1}^{n}{\bar{Q}}^{\left(k\right)}\;\;\left(z_{k}^{3}-z_{k-1}^{3}\right)$$

where n is the number of plies and *z_k−1_* and *z_k_* define the lower and upper surfaces of the *k*-th ply relative to the laminate mid-plane. The actual measured laminate thicknesses were used for the calculations. The resulting *D*-matrix coefficients were used to characterize the bending stiffness of the laminates and to support the interpretation of their different low-velocity impact responses.

The laminates were manufactured using vacuum bagging and cured in a laboratory oven in accordance with the processing recommendations for the respective material systems. The UD-3.30 and W-2.66 laminates were cured at 140 ± 5 °C for 240 min and post-cured at 180 ± 5 °C for 120 min. Processing of the previously reported UD-1.45 reference laminate is described in [29]. The temperature increased between stages at 2 °C/min, while full vacuum was maintained throughout the curing cycle. After curing, the laminate plates were cut into specimens measuring 150 mm × 100 mm using a procedure adapted from ASTM D7136/D7136M and ASTM D7137/D7137M [30,31] (Figure 1).

Low-velocity impact tests were performed using a Rosand drop-weight impact tower equipped with a hemispherical impactor with a diameter of 40 mm and a total impactor mass of 5.344 kg. All tests were conducted under controlled laboratory conditions at 21 ± 1 °C and 44 ± 2% relative humidity. Impact energies of 5 and 10 J were selected to represent two levels of impact severity and to enable evaluation of the response of the investigated thin CFRP laminates under progressively increasing impact loading. The selected impact conditions were also intended to represent low-energy impacts on thin composite skins relevant to UAV structures. These energy levels have been used in previous studies [1,11,32] to investigate the effects of laminate thickness and structural configuration on impact response and internal damage. The use of two impact energies also allows the influence of increasing impact severity on damage development and subsequent residual compressive performance to be assessed consistently across the different laminate configurations. The same absolute impact energies were applied to all configurations, although their different thicknesses resulted in impact-energy-to-thickness ratios of 3.45 and 6.90 J/mm for the 1.45 mm UD laminate, 1.52 and 3.03 J/mm for the 3.30 mm UD laminate, and 1.88 and 3.76 J/mm for the 2.66 mm woven laminate at 5 and 10 J, respectively. The standard impact-energy-to-thickness ratio of 6.7 J/mm specified in ASTM D7136/D7136M was not adopted as a common target because the study focused on two fixed low-energy impact levels relevant to thin UAV composite skins. Force–time, displacement–time, energy–time, and force–displacement responses were continuously recorded during each test.

**Figure 2:**
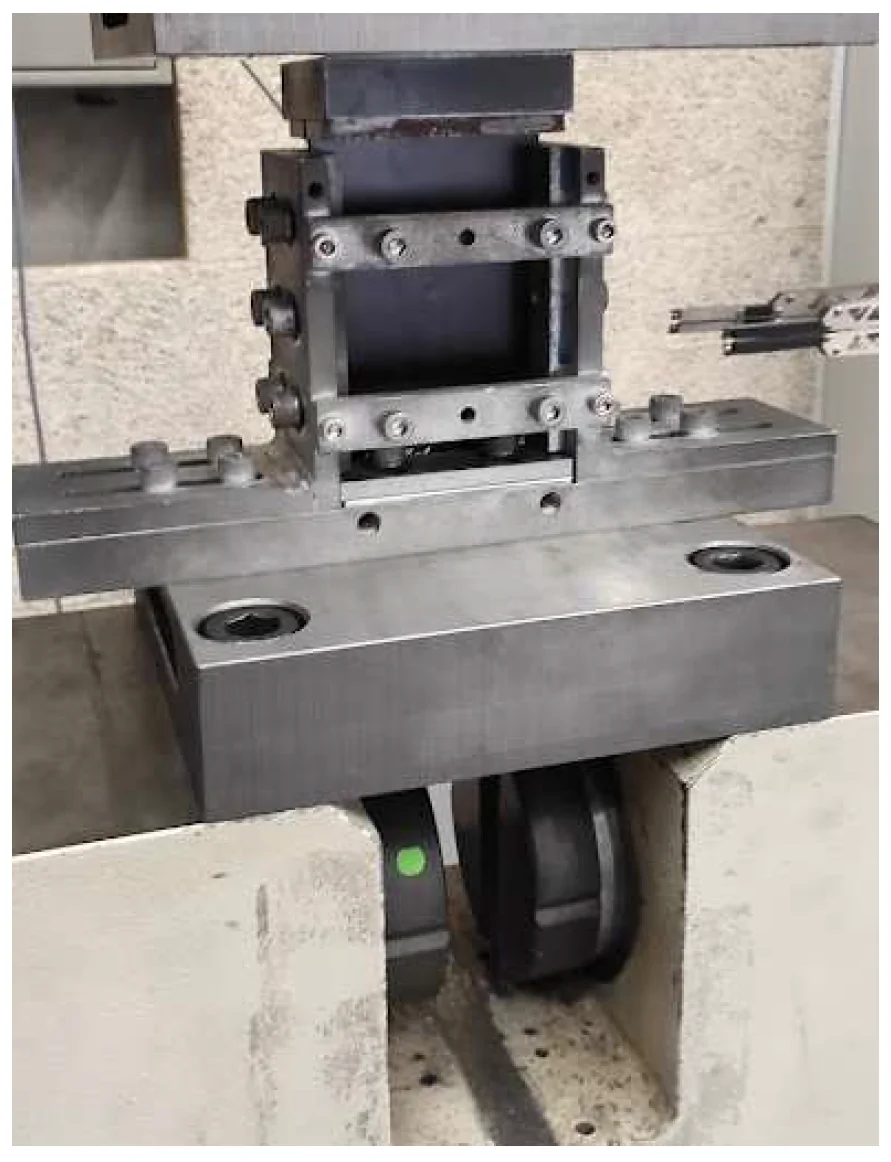
Experimental setup for compression-after-impact testing, showing the anti-buckling fixture and specimen positioning in the testing machine.

**Figure 3:**
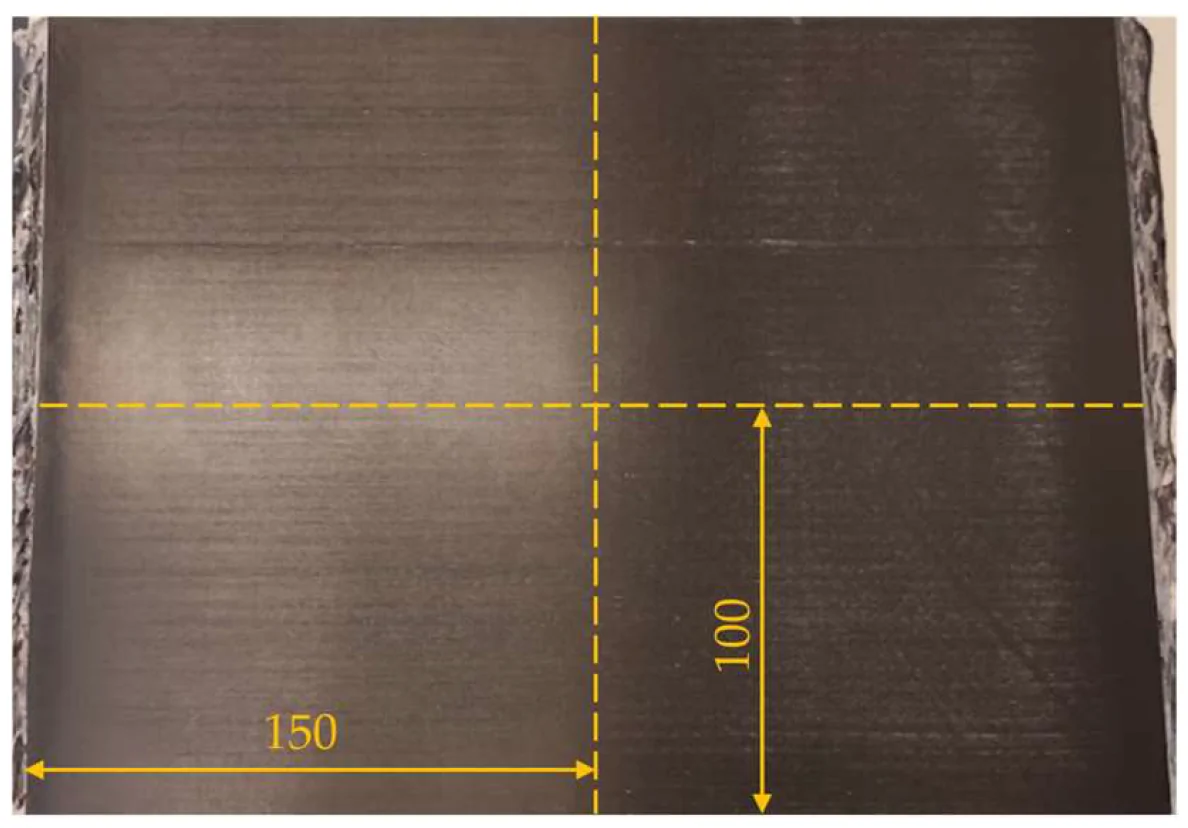
Geometry and representative specimens of the investigated CFRP laminates.

The compressive performance of the investigated laminates was evaluated in three specimen states: virgin, impacted, and post-impact healing. After impact, the specimens assigned to the healing groups for all three laminate configurations underwent thermal healing at 150 °C for 30 min in a laboratory oven [29]. During the healing treatment, the specimens were placed in the oven without additional mechanical pressure or vacuum. These conditions differed from the vacuum-assisted or pressure-assisted processes used during laminate manufacturing. Residual and post-healing compressive strength was evaluated using the CAI test configuration using a Schenck Trebel RME 600 universal testing machine. The 150 × 100 mm specimens were positioned in the anti-buckling fixture, which provided lateral support during testing. The specimen ends were also clamped to prevent local crushing under the applied compressive load (Figure 2). The tests were performed under displacement control at a crosshead rate of 1.31 mm/min. Anti-buckling fixtures were employed to prevent global instability during compression loading. The specimens were cut from laminate plates manufactured separately for each configuration, with nominal specimen dimensions of 150 mm × 100 mm (Figure 3). Independent specimens were used for the different test conditions. All three configurations were tested in the same laboratory using the same impact and CAI testing equipment and fixtures. The UD-1.45 results were previously reported in [29]. Five specimens were tested for each laminate configuration and experimental condition (n = 5). Table 3 summarizes the experimental groups and corresponding impact and healing conditions.

The impact tests were performed using a Rosand drop-weight impact testing system. The contact force, impactor velocity, displacement, and energy responses were recorded and processed using the testing system software. The displacement and energy data were consistent with numerical integration of impactor velocity over time and contact force with respect to displacement, respectively. For each test, the analyzed time interval was limited to the contact event, ending when the contact force returned to zero during unloading. The experimental responses obtained from repeated tests were averaged for each laminate configuration and impact energy.

The CAI testing procedure was adapted from ASTM D7137/D7137M [31] because the investigated laminate thicknesses (1.45–3.30 mm) were below the range typically used for standard CAI specimens. The selection of relatively thin laminates was motivated by their potential application in lightweight UAV skins. The investigation of CAI behavior in thin composite laminates has also been reported by Ghelli and Minak (2011), who examined 2.75 mm thick carbon/epoxy laminates [33], Remacha et al. (2015), who developed a dedicated testing fixture for thin laminates [34], and Francesconi et al. (2023), who investigated 2.5 mm thick Z-pinned laminates using an adapted testing arrangement [35]. In the present study, the same specimen dimensions and compression fixture were used for all three configurations to maintain consistent testing conditions.

The residual compressive strength was calculated as the maximum compressive load divided by the original cross-sectional area of the specimen. The reported values represent the mean of five measurements, with the corresponding standard deviations used to characterize the experimental scatter.

The designations CPUDV, CPUD1, CPUD2, CPUD1h, and CPUD2h for the UD-1.45 laminate are retained from the previous study [29], in which the corresponding virgin, post-impact, and post-healing experimental results were originally reported. These previously published data are included in the present study as reference data for comparison with the UD-3.30 and W-2.66 configurations investigated here.

Internal impact-induced damage was characterized using grating-based X-ray phase-contrast imaging (XPCI). Although XPCI provides absorption, refraction, and scattering (dark-field) signals, the scattering signal was selected for damage characterization because previous studies and our observations demonstrated that it provided the most informative contrast for fine-scale internal damage features [25,26,27,36,37]. In particular, the scattering images provided enhanced contrast for weakly expressed impact-induced internal heterogeneities that were difficult to distinguish in conventional absorption and refraction images.

The acquired scattering images were processed to improve the visibility of the damage-related features while preserving their spatial distribution. The images were first subjected to mild noise reduction using a 3 × 3 median filter followed by Gaussian smoothing with a standard deviation of 0.7 pixels. Low-frequency background variations were subsequently estimated by Gaussian filtering and subtracted from the denoised images to enhance local scattering contrast. This post-processing was used solely to improve the visibility of features present in the recorded scattering signal and did not introduce or reconstruct additional structural features. The processed images were therefore used for qualitative assessment of the spatial distribution and morphology of the impact-induced internal damage.

The XPCI data were used for qualitative assessment of the location and morphology of impact-induced structural changes. Quantitative segmentation of the damaged area was not performed because the scattering contrast depends not only on impact-induced changes but also on the local reinforcement architecture and its associated background signal. Consequently, a common intensity threshold could not be applied reliably across the investigated UD and woven configurations without additional calibration and validation. Manual delineation of the affected region was also avoided because it would introduce a subjective criterion into the comparison. Accordingly, the XPCI images are interpreted here qualitatively and are not used to quantify damage area or damage depth.

The compressive strength was calculated as the maximum compressive load divided by the original cross-sectional area of the specimen. For both impacted and healed specimens, the compressive-strength retention, *R_σ_*, was defined relative to the corresponding virgin laminate [8,29]:

(2) $$R_{\sigma }\;=\;\frac{\sigma_{\mathrm{CAI}}}{\sigma_{\mathrm{virgin}}}\;\;\times \;\;100\%,$$

where *σ_CAI_* denotes the compressive strength of the specimen in the considered state (*σ_CAI_* for the impacted state and *σ_CAI,healed_* for the post-impact healed state), and *σ_virgin_* is the compressive strength of the corresponding virgin laminate.

The damage index *K* was defined as:

(3) $$K\;\;=\;\;\left(1\;\;-\;\;\frac{\sigma_{\mathrm{CAI}}}{\sigma_{\mathrm{virgin}}}\right)\;\;\times \;\;100\%.$$

The healing efficiency was calculated as:

(4) $$\eta_{h}\;\;=\;\;\frac{\sigma_{\mathrm{CAI},\mathrm{healed}}\;-\;\sigma_{\mathrm{CAI}}}{\sigma_{\mathrm{virgin}}\;-\;\sigma_{\mathrm{CAI}}}\;\;\times \;\;100\%.$$

The healing efficiency, therefore, represents the fraction of the impact-induced loss in compressive strength recovered by the healing treatment. When the impact-induced strength loss was negligible, *η_h_* was not considered meaningful and was reported as *n.a.*

These parameters enable quantitative comparison of post-impact compressive-strength retention, relative strength loss, and post-healing strength recovery among the investigated laminate configurations.

Statistical significance of differences in residual compressive strength was evaluated using two-way analysis of variance (ANOVA), with laminate configuration (CPUD, UD, and CW) and test condition (virgin, 5 J impact, 10 J impact, 5 J impact followed by healing, and 10 J impact followed by healing) as factors. Each experimental group comprised five specimens (n = 5). Games–Howell post-hoc comparisons were used for selected pairwise comparisons to account for unequal variances among experimental groups. Statistical significance was defined at *p* < 0.05.

## 3. Results and Discussion

### 3.1. Low-Velocity Impact Response

**Figure 4:**
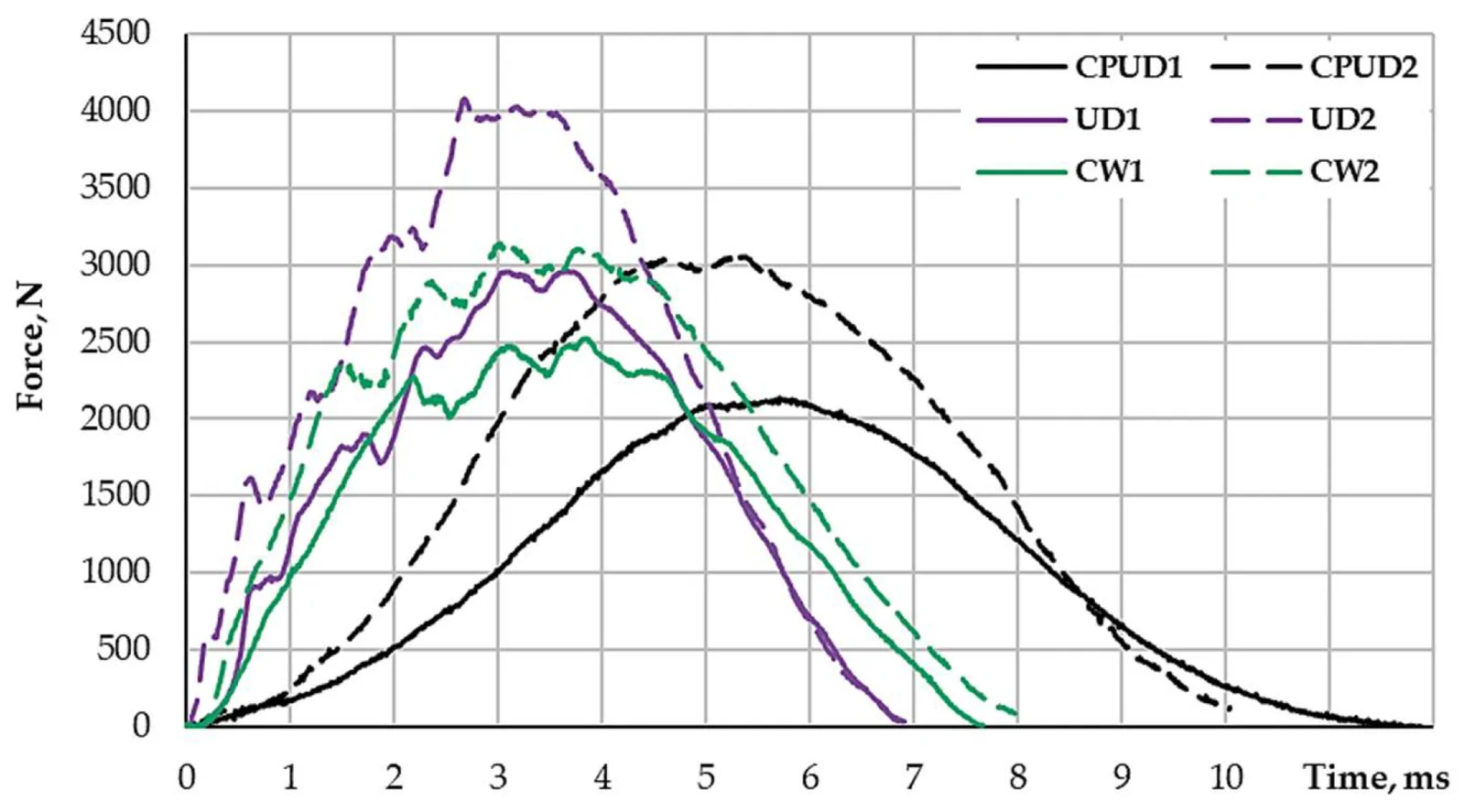
Average force–time curves of the investigated CFRP laminates at impact energies of 5 and 10 J.

**Figure 5:**
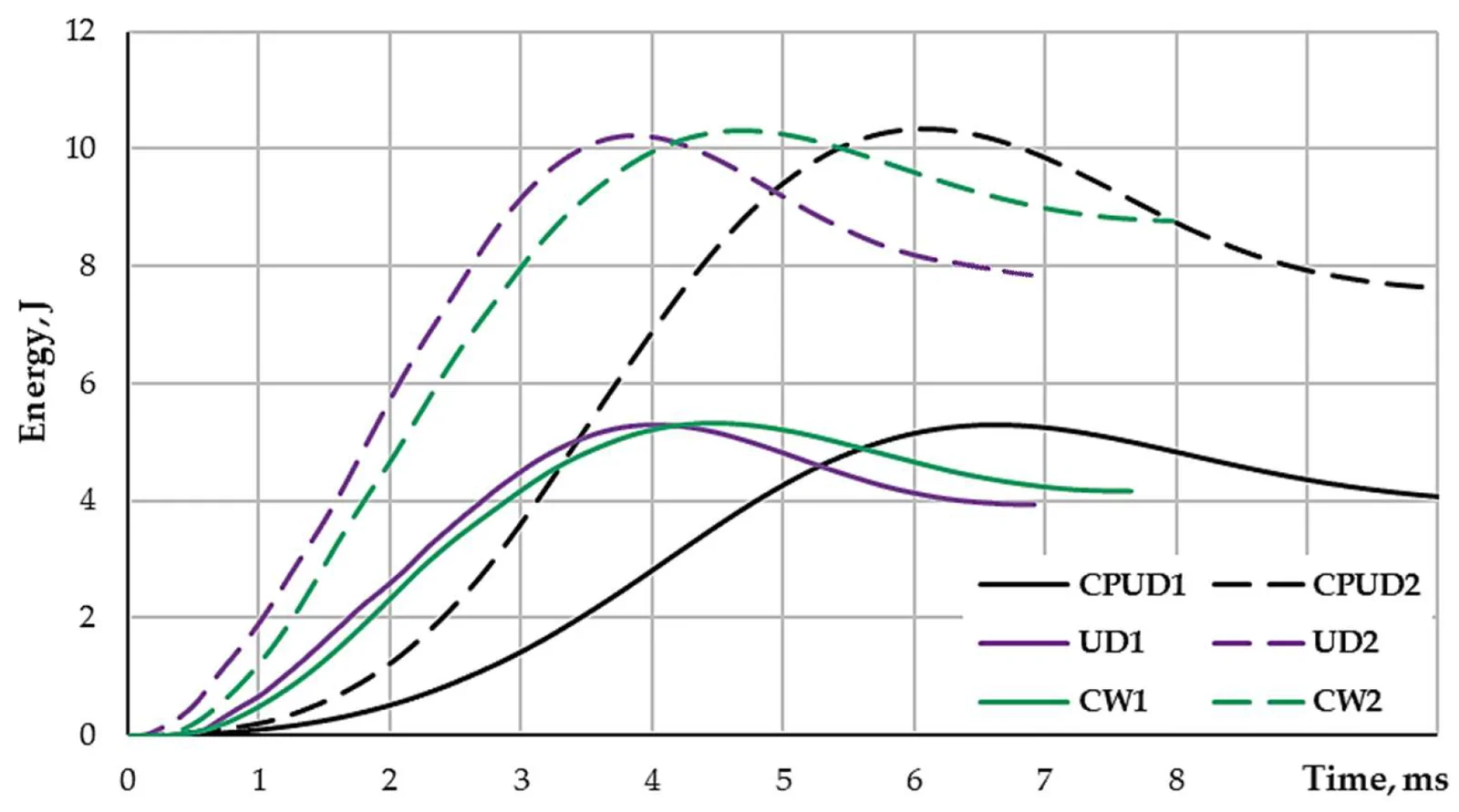
Average energy–time curves of the investigated CFRP laminates at impact energies of 5 and 10 J.

**Figure 6:**
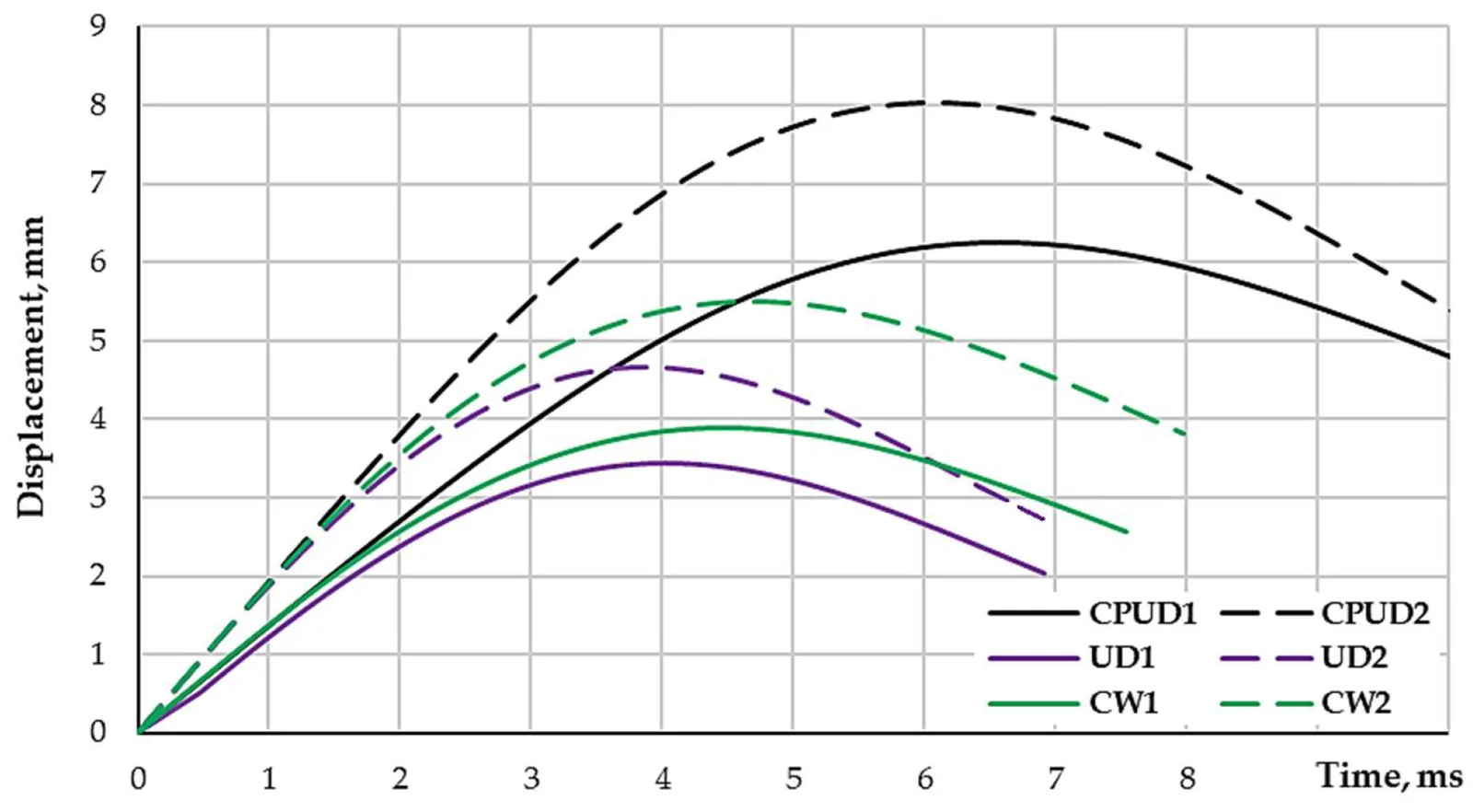
Average displacement–time curves of the investigated CFRP laminates at impact energies of 5 and 10 J.

**Figure 7:**
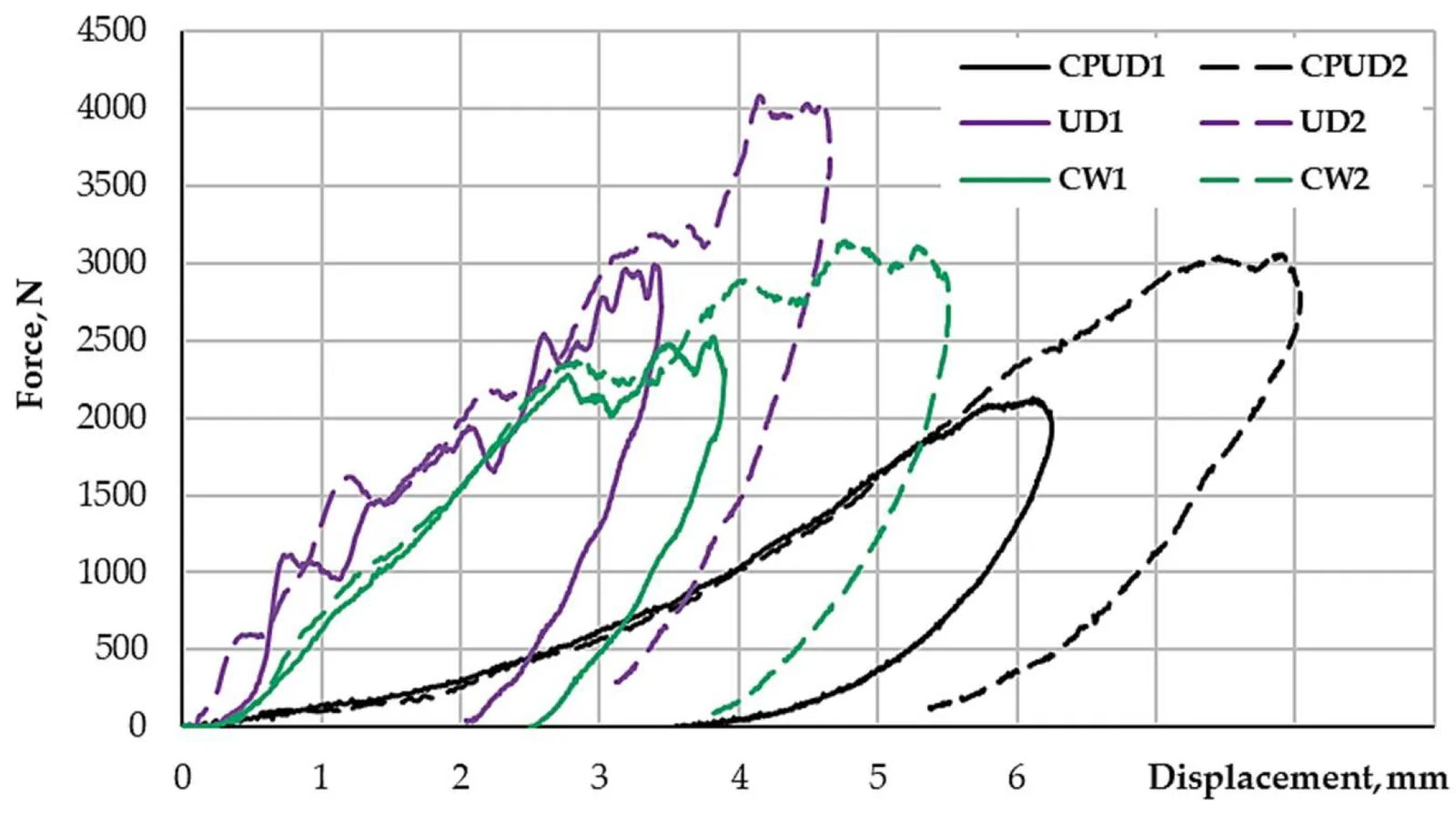
Average force–displacement curves of the investigated CFRP laminates at impact energies of 5 and 10 J.

The impact response of the investigated CFRP laminates was evaluated using force–time, energy–time, displacement–time, and force–displacement curves from drop-weight tests at impact energies of 5 and 10 J (Figure 4, Figure 5, Figure 6 and Figure 7 and Table 4). The figures show the mean response of each experimental series, while Table 4 provides the corresponding mean values, standard deviations, and coefficients of variation for the main impact parameters.

The force–time curves are presented in Figure 4. For all three laminate configurations, increasing the nominal impact energy from 5 to 10 J increased the maximum contact force, although the magnitude and shape of the response depended on the laminate configuration. At 5 J, the maximum force ranged from 2133 N for the 1.45 mm UD laminate (CPUD1) [29] to 2968 N for the 3.30 mm UD laminate (UD1), while the 2.66 mm woven laminate (CW1) exhibited an intermediate value of 2525 N. At 10 J, the corresponding maximum forces were 3053 N for CPUD2 [29], 4084 N for UD2, and 3142 N for CW2. Thus, the 3.30 mm UD laminate exhibited the highest maximum contact force at both impact energies, whereas the 1.45 mm UD laminate showed the lowest values.

The differences in the impact response can be interpreted in relation to the calculated laminate stiffness. The 1.45 mm CPUD laminate exhibited the lowest longitudinal bending stiffness (*D*_11_ = 22.6 N·m), whereas the 3.30 mm UD laminate had a substantially higher value (*D*_11_ = 272.0 N·m) (Table 2). The 2.66 mm CW laminate showed an intermediate *D*_11_ of 75.6 N·m. The much lower bending stiffness of CPUD allows greater global out-of-plane deformation, which is consistent with its lower peak contact force and broader force–time response. In contrast, the substantially higher stiffness of the UD laminate restricts global flexural deformation and promotes a steeper force increase and higher peak contact force, particularly at 10 J.

However, the results indicate that the maximum contact force cannot be directly correlated with *D*_11_ alone. Although the CW laminate had a considerably lower *D*_11_ than the UD laminate, its peak force at 5 J was only moderately lower (2525 versus 2968 N, respectively), demonstrating the contribution of other structural parameters. These include the transverse and shear bending stiffnesses (*D*_22_ and *D*_66_), laminate thickness, fiber architecture, local contact deformation and the development of impact-induced damage. The different stacking sequences determine the load-transfer paths through the laminate and influence the relative contributions of matrix cracking, fiber failure and interlaminar delamination to energy dissipation.

The shape of the force–time curves further supports this interpretation. CPUD exhibited a relatively smooth and predominantly single-peak response, whereas UD and CW showed more pronounced local maxima and force oscillations. Such features may be associated with changes in contact stiffness during progressive damage development and load redistribution between plies [1,9,10]. The force–time response therefore reflects the combined effects of the initial laminate stiffness and damage evolution rather than a single stiffness parameter. The resulting internal damage distributions are examined in greater detail using XPCI.

Increasing the impact energy from 5 to 10 J increased the maximum force by approximately 43% for the 1.45 mm UD laminate, from 2133 to 3053 N, and by approximately 38% for the 3.30 mm UD laminate, from 2968 to 4084 N. The increase for the woven laminate was smaller, approximately 24%, from 2525 to 3142 N. This indicates that the three configurations exhibited different changes in their impact-force response with increasing impact severity.

Figure 5 shows the energy–time curves. The maximum recorded energy was close to the prescribed impact energy for all configurations, ranging from 5.29 to 5.32 J at the nominal 5 J level and from 10.23 to 10.35 J at the nominal 10 J level. The small variation confirms the consistency of the applied impact conditions and provides a suitable basis for comparing the laminate responses. Although the maximum energy levels were similar, the curves evolved differently among the configurations, reflecting differences in the interaction between the impactor and laminate during unloading. These differences result from the combined effects of elastic deformation, irreversible damage, and energy dissipation within the laminate.

The displacement–time curves in Figure 6 further demonstrate the effect of laminate configuration. At 5 J, the maximum displacement reached 6.25 mm for the 1.45 mm UD laminate, compared with 3.44 mm for the 3.30 mm UD laminate and 3.90 mm for the woven laminate. At 10 J, the corresponding values were 8.04, 4.66, and 5.51 mm, respectively. Thus, the same ranking was maintained at both impact energies: 1.45 mm UD > woven > 3.30 mm UD. The maximum displacement of the thin UD laminate was about 82% higher than that of the 3.30 mm UD laminate at 5 J and about 73% higher at 10 J, confirming the much greater contribution of global deformation in the thinner laminate, consistent with its much lower bending stiffness.

Figure 7 combines the contact force and global deformation responses. The 3.30 mm UD laminate developed the highest forces over the smallest displacement range, whereas the 1.45 mm UD laminate exhibited substantially larger displacements at lower force levels. The woven laminate showed intermediate characteristics. These results demonstrate that identical nominal impact energies produced different combinations of contact force and global deformation depending on laminate configuration. Therefore, the lower maximum force of the thin laminate should not be interpreted directly as superior impact resistance, just as the higher force of the thicker laminate does not by itself establish superior damage tolerance or residual strength.

The non-zero displacement at the end of the force–displacement curves (Figure 7) results from the selected end-of-contact criterion. When the contact force returns to zero, the impactor is rebounding but has not yet returned to its initial position. Consequently, this displacement does not represent the permanent deformation of the laminate. Likewise, the non-zero energy at the end of the energy–time curves (Figure 5) represents the net energy transferred during the contact event rather than energy dissipated exclusively through material damage. Table 4 summarizes the mean impact parameters and their statistical scatter. The coefficients of variation for maximum force ranged from 1.2 to 7.2%, while those for maximum displacement ranged from 1.0 to 8.4%, indicating good repeatability of the impact measurements. The relatively small scatter in the maximum recorded energy further confirms the consistency of the applied impact conditions.

When impact energy is normalized by laminate thickness, the 1.45 mm UD configuration has the highest impact-energy-to-thickness ratio at both impact levels, followed by the 2.66 mm woven and 3.30 mm UD configurations. However, this normalization does not change the measured responses or establish a ranking of the laminates at equivalent normalized impact energies. Accordingly, the differences observed at the same absolute impact energy should be interpreted as configuration-dependent responses rather than independent effects of laminate thickness or reinforcement architecture.

Overall, the three laminate configurations exhibited distinct impact responses under the same nominal impact energies, primarily reflected in different combinations of contact force and global deformation. Higher impact energy amplified these differences but did not, by itself, directly indicate internal damage severity. Therefore, the mechanical impact response should be interpreted alongside the XPCI observations and subsequent CAI results to establish how different laminate configurations and their internal damage states affect post-impact compressive performance.

### 3.2. Qualitative Characterization of Internal Damage by XPCI

As described in Section 2, the XPCI results are interpreted qualitatively because the architecture-dependent scattering background does not allow a common segmentation threshold to be applied reliably across all investigated configurations without additional calibration and validation. Therefore, differences in the apparent size or intensity of XPCI features are not interpreted quantitatively as differences in damage area or severity.

**Figure 8:**
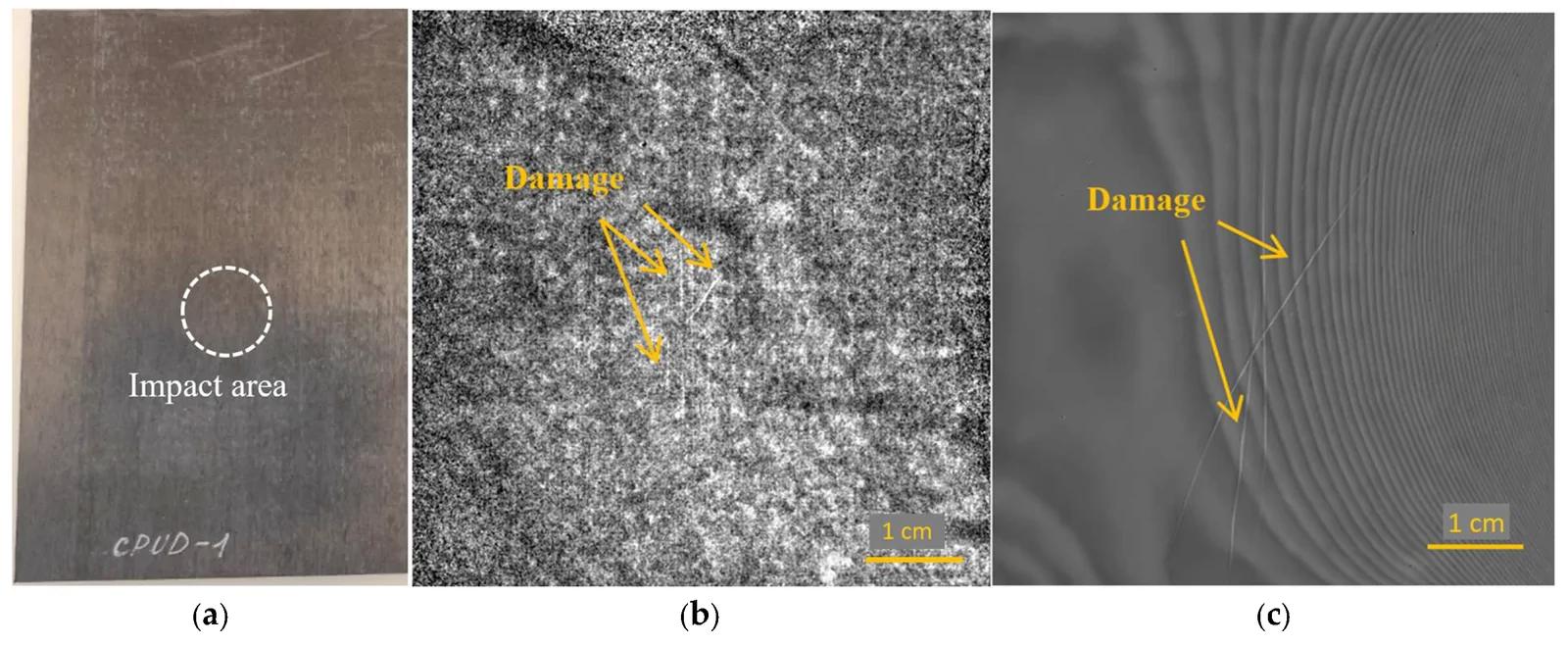
Visual photograph (**a**), scattering-mode XPCI image (**b**) and processed XPCI image (**c**) of the 1.45 mm UD laminate after a 5 J impact (CPUD1). Adapted from [29] under the terms of the Creative Commons Attribution (CC BY 4.0) license.

**Figure 9:**
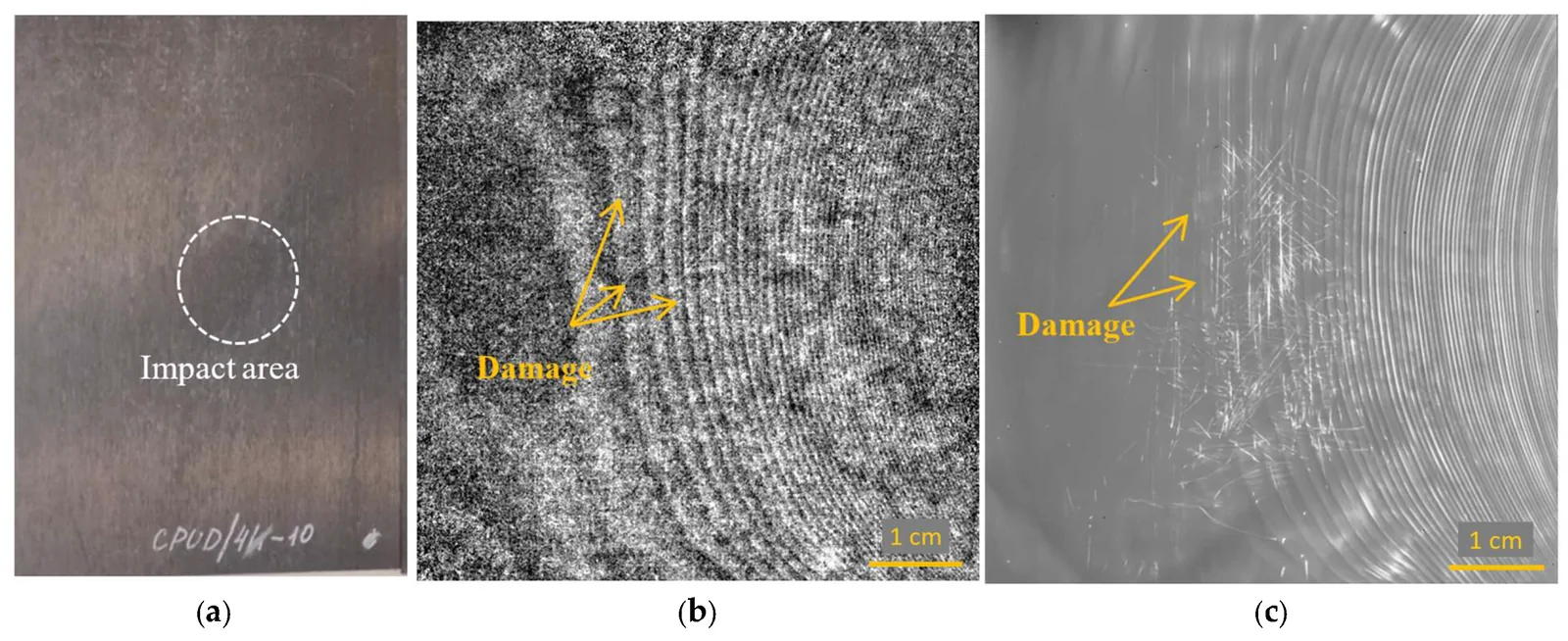
Visual photograph (**a**), scattering-mode XPCI image (**b**) and processed XPCI image (**c**) of the 1.45 mm UD laminate after a 10 J impact (CPUD2). Adapted from [29] under the terms of the Creative Commons Attribution (CC BY 4.0) license.

**Figure 10:**
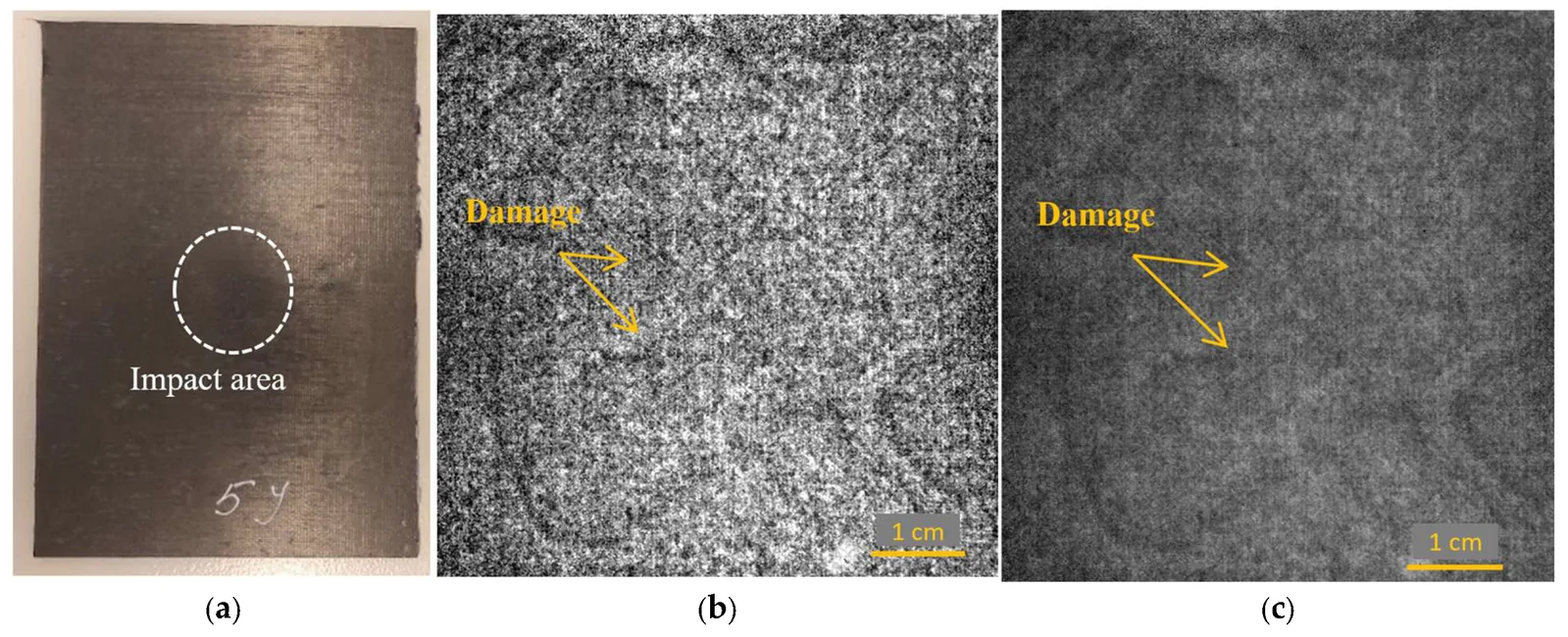
Visual photograph (**a**), scattering-mode XPCI image (**b**) and processed XPCI image (**c**) of the 3.30 mm UD laminate after a 5 J impact (UD1).

**Figure 11:**
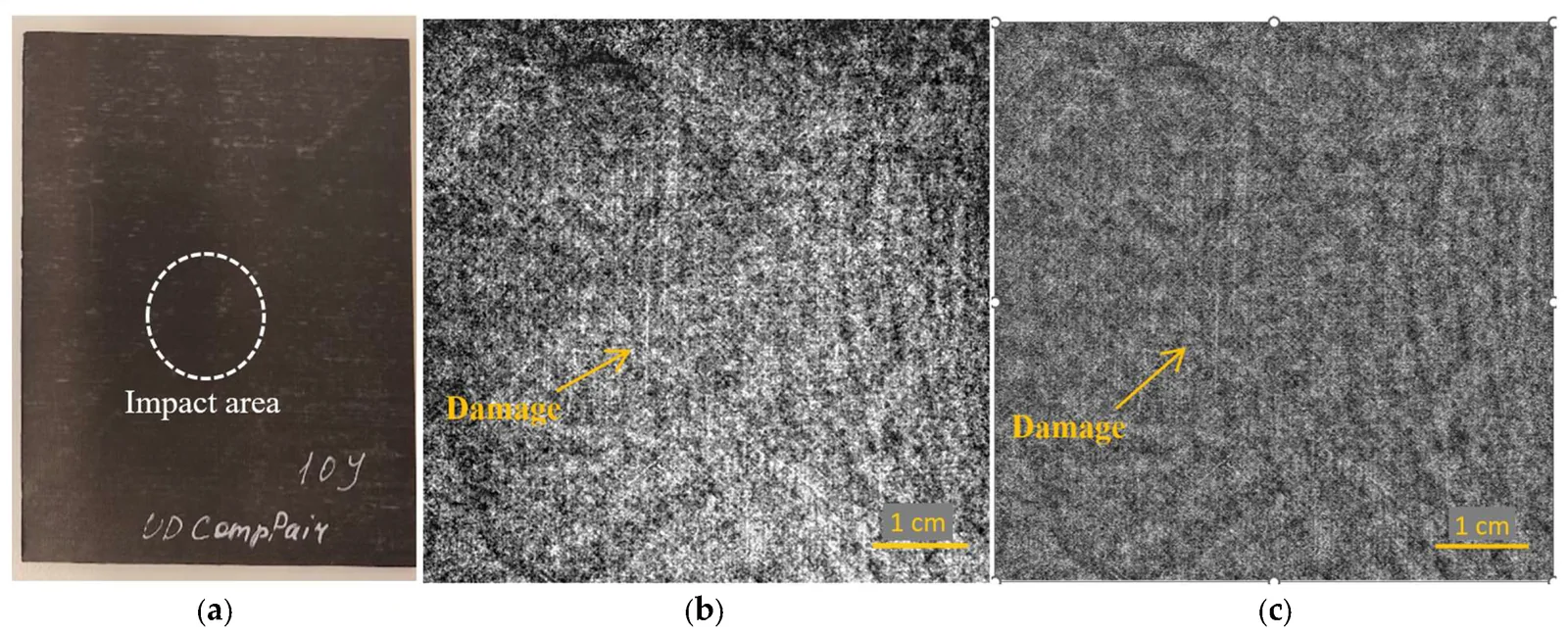
Visual photograph (**a**), scattering-mode XPCI image (**b**) and processed XPCI image (**c**) of the 3.30 mm UD laminate after a 10 J impact (UD2).

**Figure 12:**
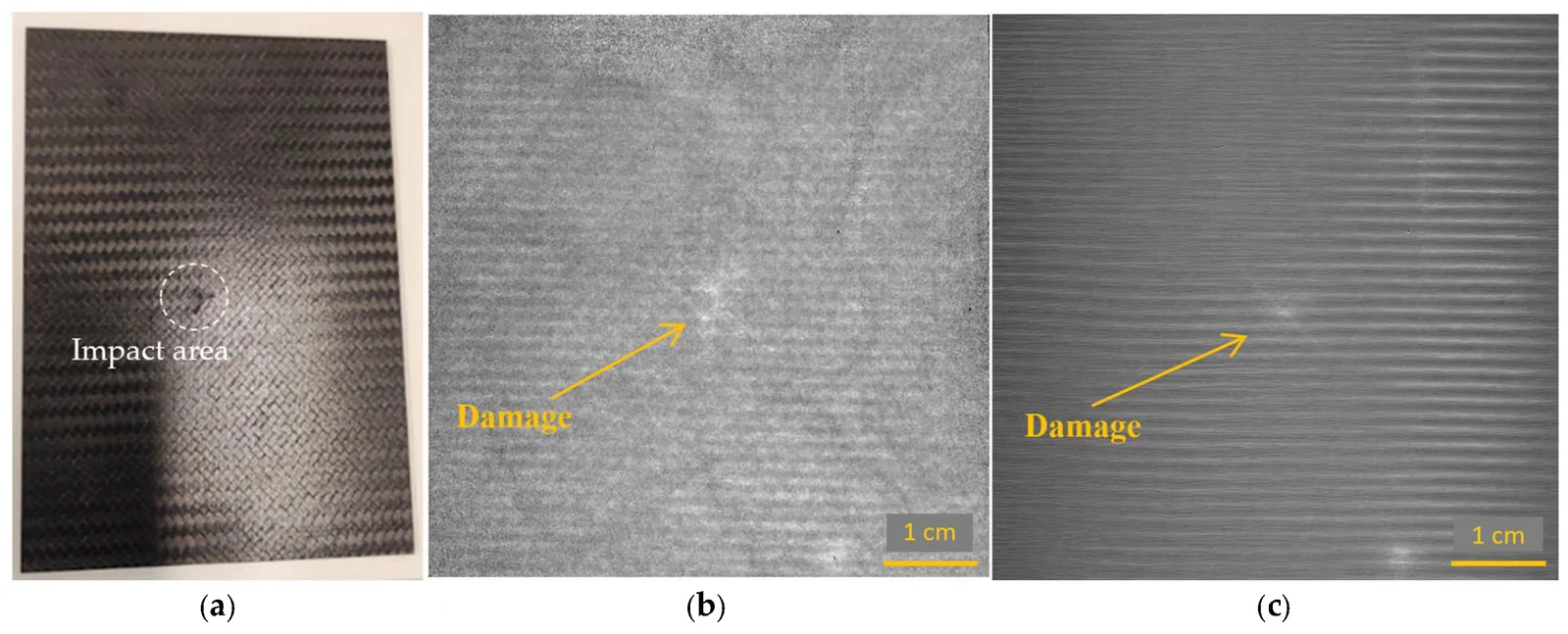
Visual photograph (**a**), scattering-mode XPCI image (**b**) and processed XPCI image (**c**) of the 2.66 mm woven CFRP laminate after a 5 J impact (CW1).

**Figure 13:**
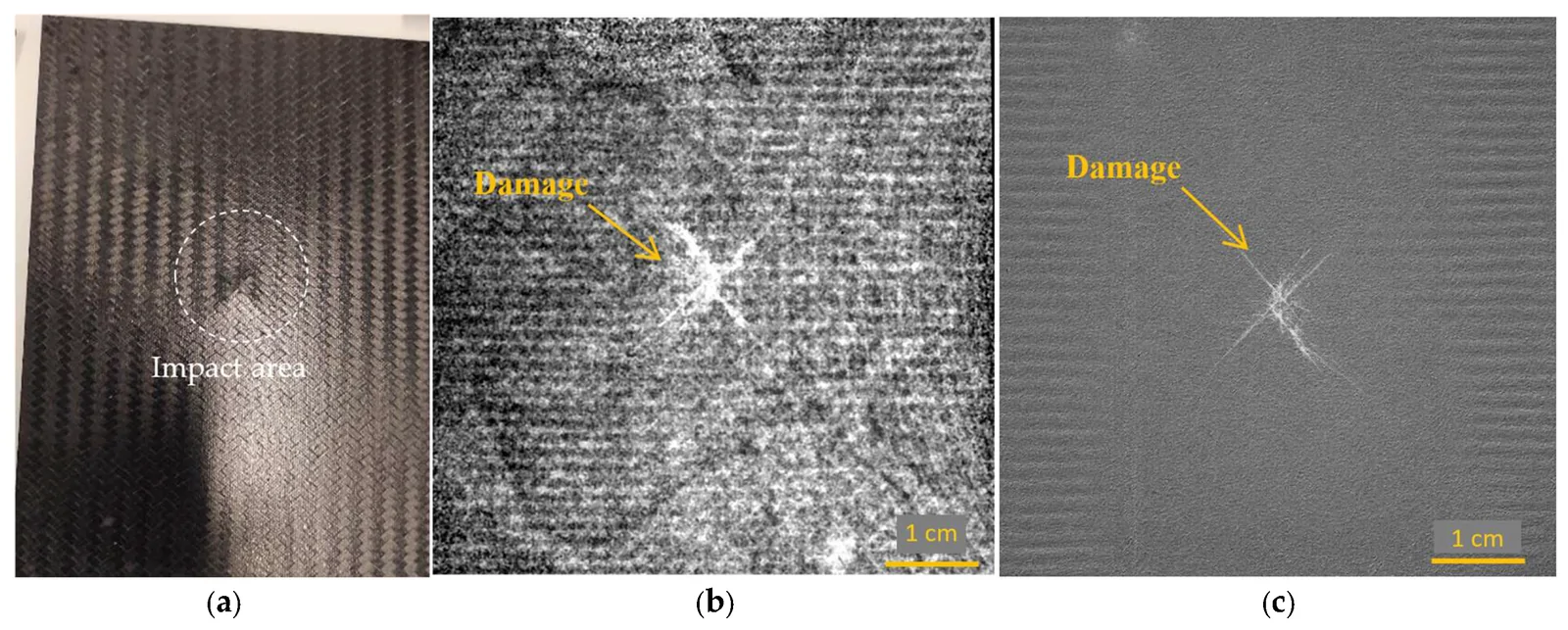
Visual photograph (**a**), scattering-mode XPCI image (**b**) and processed XPCI image (**c**) of the 2.66 mm woven CFRP laminate after a 10 J impact (CW2).

Internal damage was characterized qualitatively by visual inspection and grating-based X-ray phase-contrast imaging, as shown in Figure 8, Figure 9, Figure 10, Figure 11, Figure 12 and Figure 13. The XPCI images were acquired with a spatial sampling of 50 µm/px. The Moiré patterns (banding) visible in the images originate from the X-ray grating interferometer and are therefore instrumental features rather than indications of material damage. The analysis focused on the location and morphology of impact-induced internal changes within each laminate configuration and on their qualitative evolution between the 5 J and 10 J impact conditions. Particular attention was given to the scattering-mode images because of their sensitivity to local microstructural heterogeneities and impact-induced internal changes. No quantitative measurements of damage area, damage depth, or XPCI signal intensity were performed. Accordingly, the XPCI results were used to qualitatively characterize the observed internal damage patterns and to relate these observations to the mechanical impact response discussed in Section 3.1.

The XPCI results for the 1.45 mm UD laminate were previously reported in [29] and are included here as reference data for comparison with the other two laminate configurations. For the 1.45 mm UD laminate impacted at 5 J, the contact region on the surface of specimen CPUD1 remained only weakly visible (Figure 8a), whereas the scattering-mode XPCI image revealed a localized region of internal structural disturbance around the impact location (Figure 8b). Thus, the limited surface indication did not fully represent the internal response, consistent with the characteristic nature of barely visible impact damage in CFRP laminates at relatively low impact energies [22,23,24].

At 10 J, specimen CPUD2 exhibited more pronounced XPCI contrast around the impact location (Figure 9b), while the surface indication (Figure 9a) remained relatively limited. The relatively large deformation of this thin laminate during impact indicates a greater contribution of global bending to its overall impact response. The corresponding XPCI images show localized internal changes around the impact region; however, their apparent extent and intensity are not interpreted quantitatively.

The 3.30 mm UD laminate exhibited a different XPCI response. Following the 5 J impact, the XPCI images showed localized or diffuse changes around the impact region (Figure 10). At the same impact energy, the 3.30 mm laminate exhibited a higher maximum force of 2968 N and a smaller maximum displacement of 3.44 mm. At 10 J, the affected region stayed less clearly distinguishable in the XPCI image (Figure 11), while the maximum force increased to 4084 N and displacement to 4.66 mm. Thus, the two UD configurations exhibited different internal responses despite being subjected to the same nominal impact energies. The affected region in the 3.30 mm laminate was less clearly delineated in the XPCI images, indicating that differences in scattering contrast should not be interpreted directly as differences in damage severity or damage area.

Comparing the two UD laminates also illustrates why maximum contact force alone is insufficient to characterize the impact response. To account for different laminate thicknesses and deformation levels, we also considered the product F_max_d_h_/t (where F_max_—maximum force in impact, d_h_—maximum displacement in impact and t—thickness of samples (Table 3)), as an approximate normalized response parameter. At 5 J, this parameter was 9.2 kJ/m for the 1.45 mm laminate and 3.1 kJ/m for the 3.30 mm laminate; at 10 J, the corresponding values were 16.9 and 5.8 kJ/m, respectively. Thus, although the thicker laminate sustained substantially higher maximum forces, its smaller deformation relative to thickness resulted in a lower value of F_max_d_h_/t. This difference reflects the distinct force–deformation regimes of the two laminates. However, F_max_d_h_/t should be regarded only as a comparative, approximate descriptor rather than a direct measure of damage or energy dissipation, since the deformation and stress fields in heterogeneous composite laminates are highly non-uniform.

For the 2.66 mm woven laminate, the visual, scattering-mode, and processed XPCI images obtained after the 5 J impact are shown in Figure 12 and after the 10 J impact in Figure 13. In contrast to the UD laminates, the impact-induced changes are visible as a localized change in surface appearance around the impact region (Figure 12a and Figure 13a). The XPCI images likewise show a localized region of altered contrast around the impact site. However, the characteristic background signal associated with the woven reinforcement architecture limits precise identification and delineation of the affected region. Nevertheless, qualitatively different spatial patterns of impact-induced XPCI contrast were observed in the woven and UD configurations. Because the scattering background itself depends on reinforcement architecture, the apparent extent and intensity of these features should not be interpreted quantitatively as measures of damage area or severity. Accordingly, the present observations are limited to qualitative differences in the spatial distribution and morphology of the XPCI contrast.

Across all three configurations, increasing the impact energy from 5 to 10 J resulted in a more pronounced internal structural response in the XPCI images. At the same time, the magnitude and character of the response differed between the laminates. The 1.45 mm UD laminate combined the lowest contact forces with the largest global displacements, whereas the 3.30 mm UD laminate exhibited the highest forces and smallest displacements. The woven laminate showed intermediate mechanical behavior. The force–time and XPCI results can be considered together, particularly for the 3.30 mm UD and woven laminates, which exhibited more pronounced force fluctuations than the 1.45 mm UD laminate. The XPCI images confirmed internal structural changes in these configurations, but the present measurements do not provide sufficient temporal or quantitative information to associate individual force peaks with specific mechanisms such as matrix cracking, delamination, or fiber damage.

Overall, the XPCI observations complement the global impact measurements by showing that differences in force–deformation response are accompanied by differences in the internal structural response of the laminates. In particular, limited surface indications may coexist with localized subsurface changes, emphasizing the value of internal imaging for evaluating impact damage in CFRP laminates. The observed configuration-dependent damage patterns provide a basis for examining their relationship with residual compressive performance in the subsequent CAI results.

### 3.3. Compression-After-Impact Response and Residual Strength

**Figure 14:**
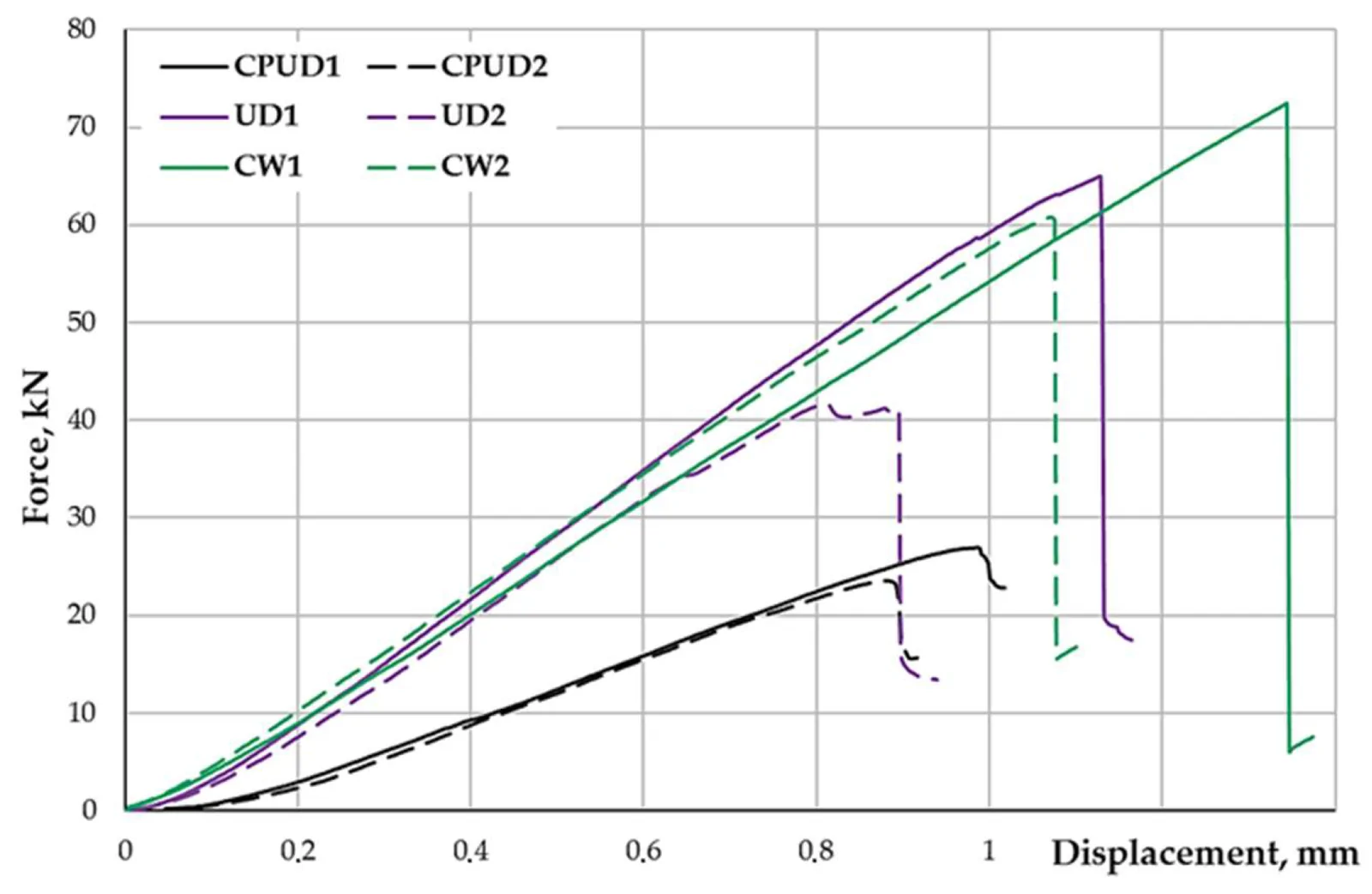
Representative load–displacement curves obtained during compression-after-impact testing of CFRP laminates.

**Figure 15:**
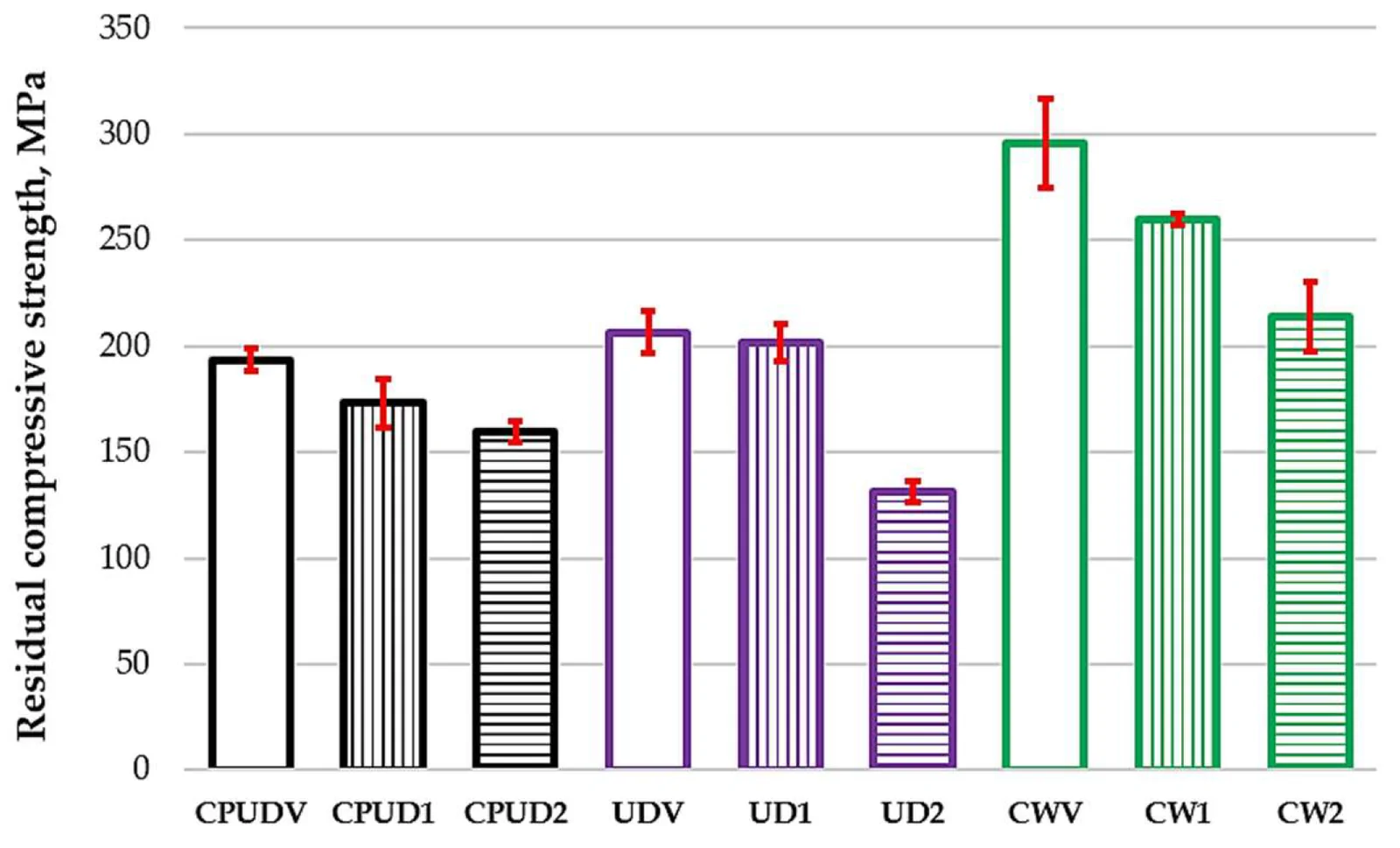
Residual compressive strength of the investigated CFRP laminates under different impact conditions. Error bars represent the standard deviation.

Compression-after-impact testing evaluated how impact-induced damage affected the subsequent load-carrying capacity of the investigated laminates. Representative load–displacement curves are shown in Figure 14, while the mean residual compressive strength values and corresponding statistical parameters are summarized in Table 5 and Figure 15. For the impacted specimens, the load–displacement curves were initially close to linear, then became nonlinear as the load approached the maximum, followed by a rapid decrease in load associated with loss of load-carrying capacity.

The compressive strength results for the 1.45 mm UD laminate were previously reported in [29] and are included here for comparative purposes. For the 1.45 mm UD laminate, the mean compressive strength of the unimpacted reference specimens (CPUDV) was 193.63 ± 5.0 MPa. Following the 5 J impact, the residual strength decreased to 173.11 ± 11.23 MPa, while the 10 J impact reduced it further to 159.41 ± 5.09 MPa [29]. The corresponding retained-strength ratios *R_σ_* (Equation (2) were 89 and 82%, respectively, corresponding to damage indices *K* (Equation (3)) of 11 and 18%. Thus, the mean residual compressive strength decreased with increasing impact energy. The 3.30 mm UD laminate exhibited a markedly different CAI response. Its unimpacted compressive strength was 206.49 ± 10.02 MPa and decreased only slightly to 201.68 ± 8.68 MPa after the 5 J impact, corresponding to a retained strength of approximately 98%. After the 10 J impact, however, the residual strength decreased sharply to 131.53 ± 4.93 MPa, corresponding to approximately 64% strength retention and a damage index of 36%. This pronounced change occurred despite the high impact stiffness of this configuration. The 3.30 mm UD laminate exhibited the highest maximum contact force and smallest displacement at both impact energies, but this response did not translate directly into greater retention of compressive strength at 10 J. The XPCI images likewise showed an internal structural response at 10 J. Although the available data do not allow a specific damage threshold or failure mechanism to be identified, the combined results indicate that the higher impact energy produced internal changes that had a substantially greater effect on subsequent compressive strength than for the UD laminate of 1.45 mm thickness.

The pronounced reduction in strength retention of the 3.30 mm UD laminate from approximately 98% at 5 J to 64% at 10 J may be associated with the development of internal damage that is not readily distinguishable in the XPCI images. In particular, impact-induced delamination and matrix cracking could promote local instability of the damaged plies during subsequent compressive loading, resulting in a substantial reduction in load-carrying capacity. The load plateau observed at approximately 41 kN in Figure 14 may indicate progressive damage development or local instability before final failure. However, these mechanisms remain possible explanations rather than experimentally confirmed failure modes, since additional investigations such as micro-CT, ultrasonic C-scanning, or cross-sectional examination were not performed.

The 2.66 mm woven laminate exhibited the highest absolute compressive strength both before and after impact. Its unimpacted strength was 295.86 ± 21.07 MPa, decreasing to 259.76 ± 2.99 MPa after 5 J and 213.98 ± 16.35 MPa after 10 J. The corresponding retained-strength ratios were 88 and 72%, with damage indices of 12 and 28%, respectively. Thus, the mean residual compressive strength decreased with increasing impact energy, following the same overall trend observed for the UD laminates. This behavior is consistent with the localized internal damage observed by XPCI and the intermediate force–displacement response of the woven laminate. When comparing the three configurations, although the woven laminate maintained the highest absolute residual compressive strength after 10 J, the ranking changed when strength was normalized to the corresponding unimpacted values: the 1.45 mm UD laminate retained 82%, the woven laminate 72%, and the 3.30 mm UD laminate 64% of their initial strengths. This comparison highlights that absolute residual strength and normalized strength retention describe different aspects of post-impact performance. The former reflects the load-carrying capacity remaining after impact, whereas the latter indicates the proportion of the original mechanical performance preserved. Therefore, the higher absolute residual strength of the woven laminate should not be interpreted directly as superior damage tolerance, as its initial compressive strength was also higher.

Two-way ANOVA showed significant effects of laminate configuration (F (2,60) = 418.37, *p* < 0.001), test condition (F (4,60) = 81.35, *p* < 0.001), and their interaction (F (8,60) = 7.67, *p* < 0.001) on residual compressive strength. The significant interaction indicates that the effect of impact and subsequent healing depended on the laminate configuration.

Games–Howell post-hoc comparisons further showed that the reduction in compressive strength after the 10 J impact was significant relative to the virgin condition for all three configurations (CPUD: *p* < 0.001; UD: *p* < 0.001; CW: *p* = 0.001). In contrast, the differences between the virgin and 5 J impacted conditions were not statistically significant (CPUD: *p* = 0.057; UD: *p* = 0.920; CW: *p* = 0.079).

Post-hoc comparisons between laminate configurations showed that CW differed significantly from both CPUD and UD under all test conditions (*p* < 0.01). CPUD and UD did not differ significantly in the virgin condition (*p* = 0.095), but significant differences between these two configurations were observed after the 5 J impact (*p* = 0.006), 10 J impact (*p* < 0.001), 5 J impact followed by healing (*p* = 0.049), and 10 J impact followed by healing (*p* = 0.041).

**Figure 16:**
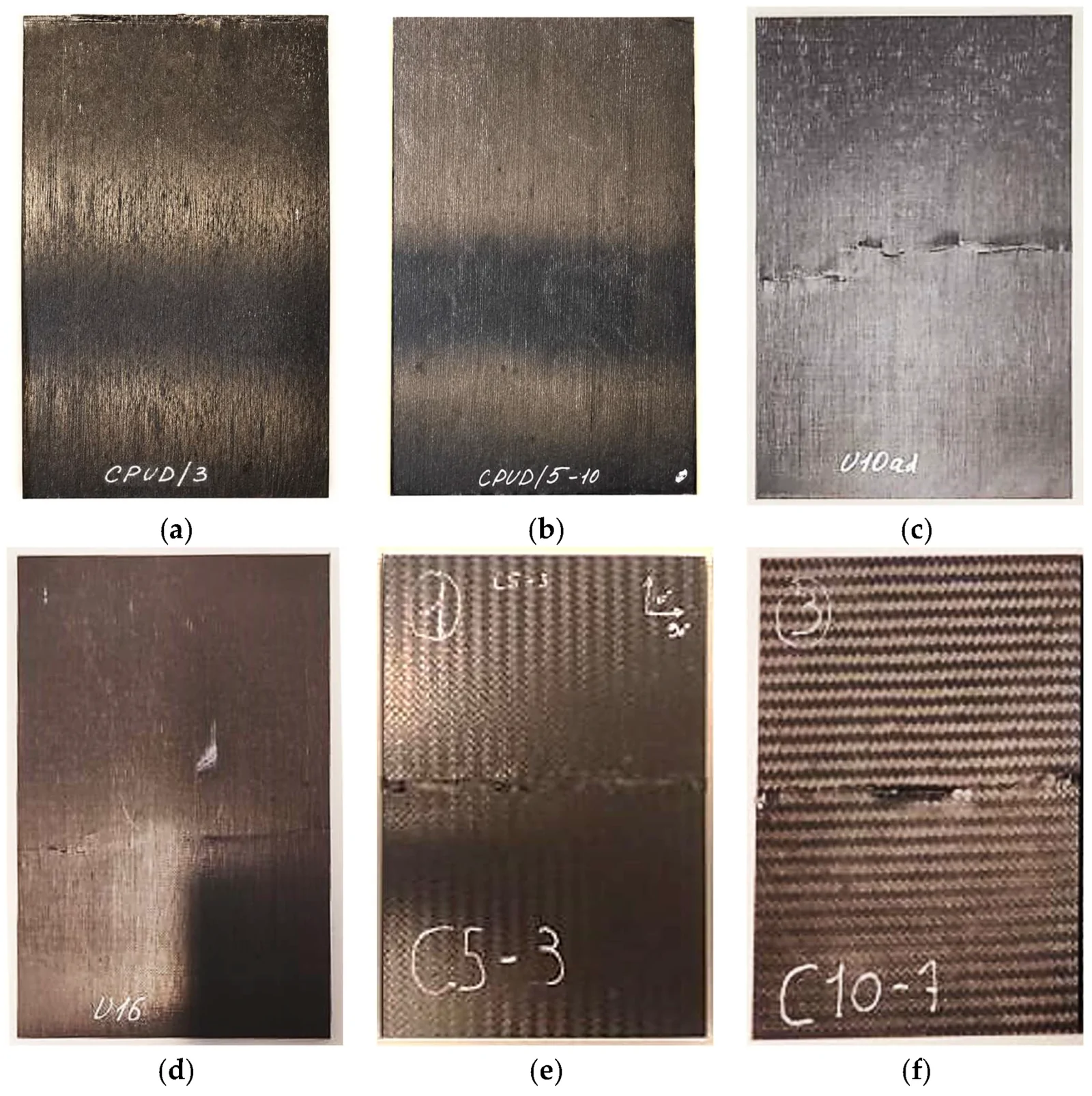
Representative post-CAI appearance of the investigated CFRP specimens after prior low-velocity impact: (**a**,**b**) CPUD1 and CPUD2, respectively; (**c**,**d**) UD1 and UD2, respectively; (**e**,**f**) CW1 and CW2, respectively.

Representative post-CAI appearances of the specimens are shown in Figure 16. The macroscopic failure patterns differed markedly among the laminate configurations. The UD-1.45 specimens predominantly exhibited end-region compressive failure (CAB), whereas lateral failure in the middle region (LDM) was observed in the UD-3.30 and W-2.66 laminates. For the thinnest laminate, failure near the specimen ends is not unexpected, considering its reduced thickness and the limitations associated with applying a conventional CAI fixture to thin laminates. The CAB-type failures were included in the calculation of the reported mean compressive strengths. However, because these failures occurred away from the central impact-damaged region, the measured strengths were not necessarily governed by the impact damage itself. Consequently, the UD-1.45 results should be interpreted as apparent compressive strengths obtained using an adapted CAI procedure rather than as fully standard-compliant CAI properties. Since the photographs were obtained after compression testing, the observed failure patterns reflect the combined effects of prior impact damage and subsequent compressive loading.

Overall, the CAI results showed substantial configuration-dependent differences in residual compressive strength loss following impact. The 1.45 mm UD laminate showed a progressive reduction in retained strength with increasing impact energy; the 3.30 mm UD laminate exhibited a particularly pronounced loss of strength at 10 J despite its high impact stiffness; and the woven laminate maintained the highest absolute residual strength but not the highest normalized retention. These results confirm that impact stiffness, internal damage response, and residual compressive performance represent related but distinct aspects of laminate damage tolerance. The following section examines whether thermal healing can recover the observed impact-induced loss in compressive strength.

### 3.4. Post-Impact Healing and Recovery of Compressive Strength

The post-impact healing response of the investigated laminate configurations was evaluated by comparing the CAI strength of impacted and post-impact-healed specimen groups. The previously reported CPUD results [29] were used as reference data for comparison with the UD-3.30 and W-2.66 configurations investigated in the present study.

**Figure 17:**
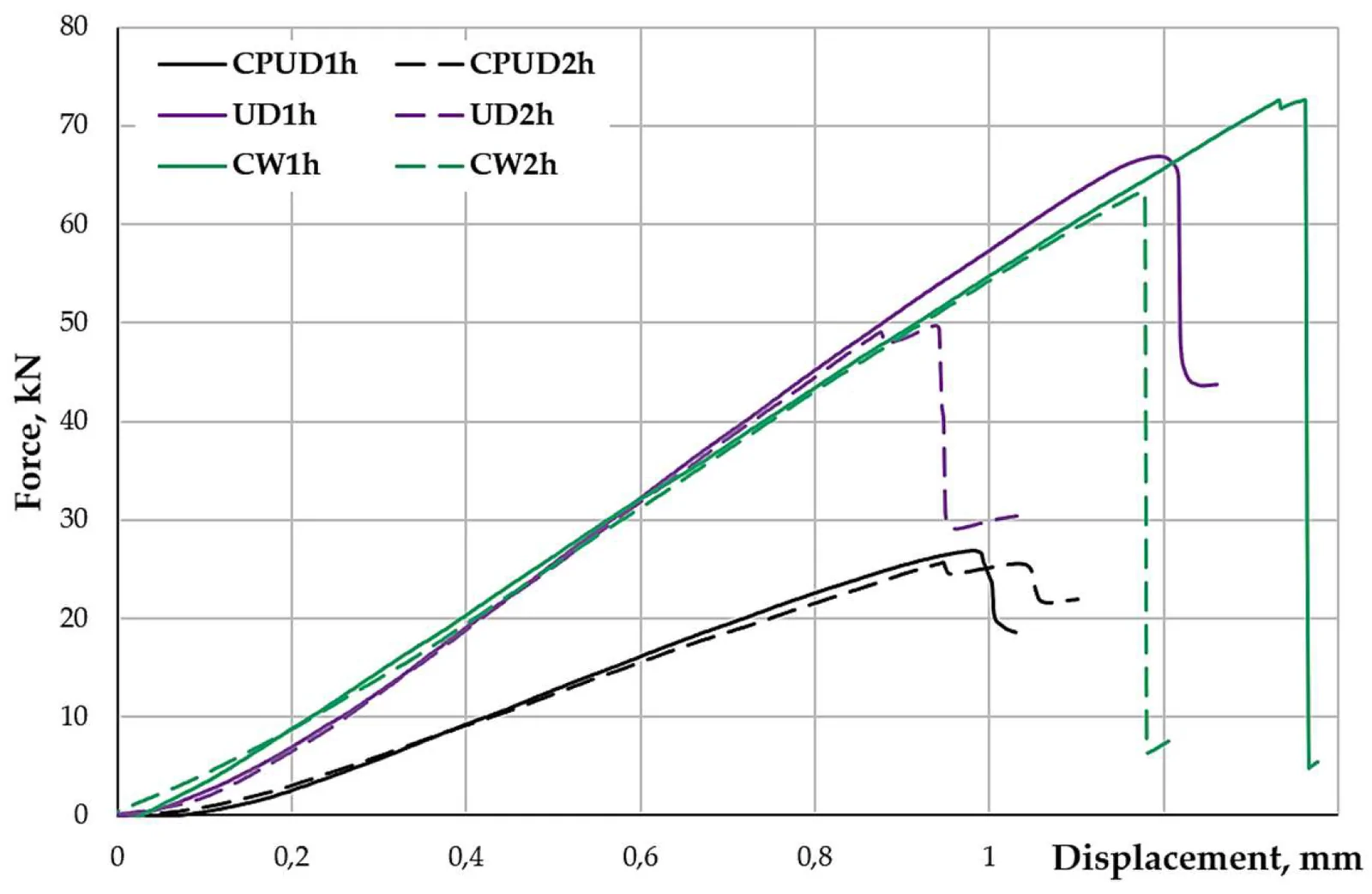
Representative load–displacement curves obtained during CAI testing of post-impact healed CFRP laminates; 1 and 2 denote prior impact energies of 5 and 10 J, respectively.

**Figure 18:**
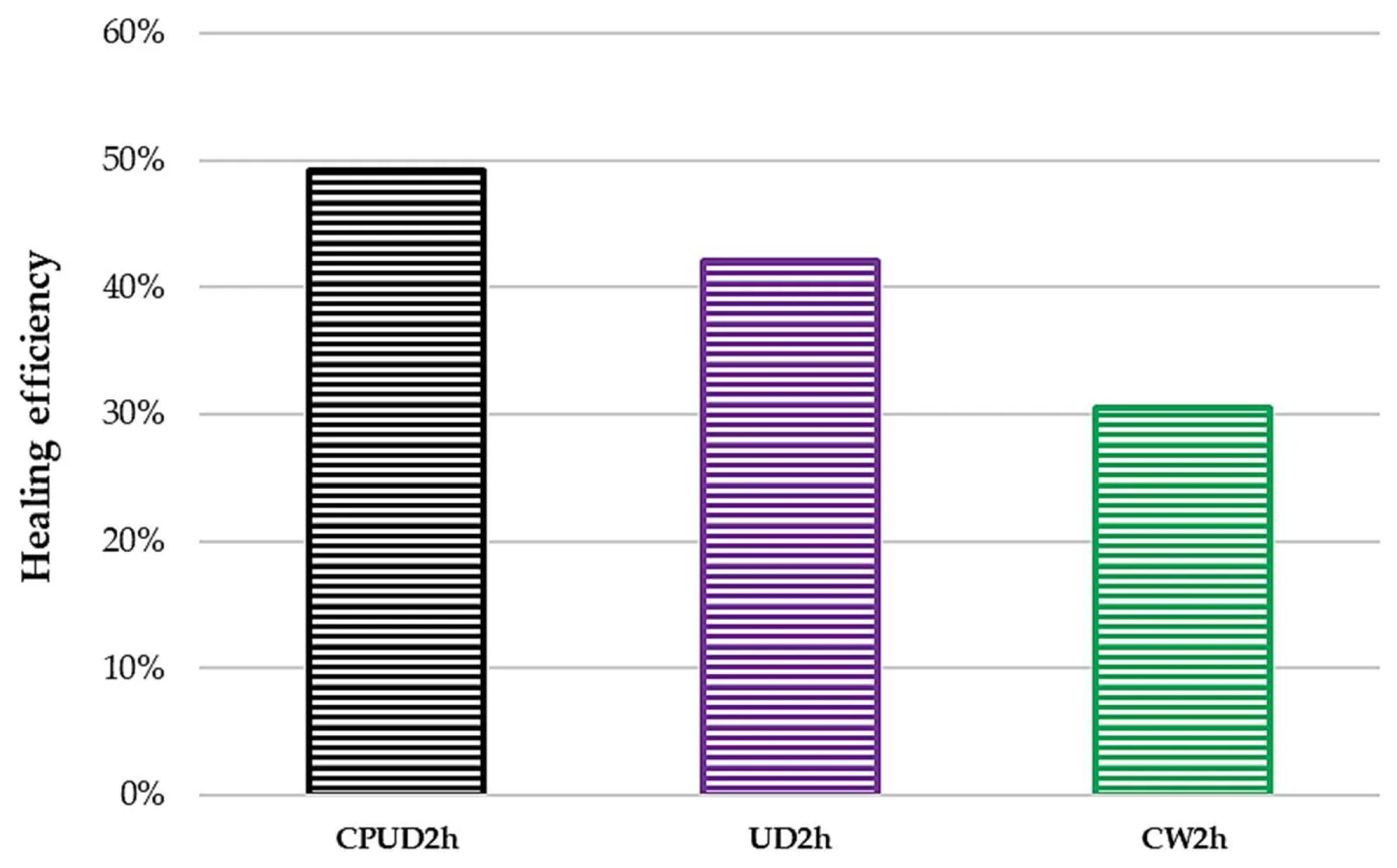
Healing efficiency of the investigated CFRP laminate configurations after 10 J impacts.

Representative load–displacement curves obtained during CAI testing of the healed specimens are shown in Figure 17. The curves exhibited an initially near-linear response, followed by nonlinear behavior approaching the maximum load and a rapid post-peak decrease. For all three configurations, specimens previously impacted at 10 J reached lower maximum loads than those impacted at 5 J, with the largest difference observed for the 3.30 mm UD laminate. The corresponding post-healing CAI strength values and statistical parameters are summarized in Table 6, while Figure 18 presents the healing efficiency at 10 J impact energy.

For the 1.45 mm UD laminate, the previously reported CPUD1h and CPUD2h results [29] showed substantial recovery at both impact energies. After 5 J impact and subsequent healing, the mean CAI strength reached 190.74 ± 5.59 MPa, corresponding to 99% of the virgin strength and a healing efficiency of 86%. After 10 J, the healed CAI strength was 176.27 ± 7.64 MPa, corresponding to 91% of the virgin value, with a healing efficiency of 49%. Thus, higher impact energy resulted in a lower final strength and a smaller recoverable fraction of the impact-induced strength loss.

The 3.30 mm UD laminate showed limited scope to calculate recovery efficiency after the 5 J impact because its pre-healing CAI strength was already close to the virgin value. Following healing, the mean strength reached 201.65 ± 6.37 MPa, corresponding to 98% strength retention. Because the impact-induced strength loss was relatively small and the post-healing strength approached the virgin value, the healing efficiency calculated using Equation (4) was not considered a meaningful basis for comparison and is reported as n.a. in Table 6. After 10 J, however, healing increased the CAI strength from 131.53 ± 4.93 MPa to 163.11 ± 6.04 MPa. Corresponding strength retention increased from 64% to 79%, with a healing efficiency of 42%, demonstrating partial recovery of the impact-induced strength loss.

The 2.66 mm woven laminate showed substantial recovery after the 5 J impact. Its post-healing CAI strength reached 294.19 ± 22.95 MPa, corresponding to 99% of the virgin strength. As the remaining strength loss was small, the healing efficiency is reported as n.a. in Table 6 rather than being used for quantitative comparison. After 10 J, the healed strength was 238.96 ± 12.67 MPa, corresponding to 81% strength retention and a healing efficiency of 31%. Thus, although substantial recovery was achieved at both impact energies, the fraction of the initial strength loss recovered was markedly lower after the 10 J impact. XPCI examination was not performed after thermal healing; therefore, the recovery of internal damage morphology could not be directly assessed in the present study.

Overall, healing efficiency was compared quantitatively only for the 10 J impact condition (Figure 18), because the small strength loss following the 5 J impact made this parameter unsuitable for evaluating recovery and could result in calculated values exceeding 100%. At 10 J, the corresponding values were 49%, 42%, and 31% for the 1.45 mm UD, 3.30 mm UD, and woven laminates, respectively. These results indicate that the fraction of impact-induced compressive-strength loss recovered through healing depended on laminate configuration, with the 1.45 mm UD laminate exhibiting the highest healing efficiency under the more severe impact condition.

Following healing after the 5 J impact, none of the three configurations differed significantly from their respective virgin conditions (CPUD: *p* = 0.903; UD: *p* = 0.884; CW: *p* > 0.999). These results indicate that the post-healing CAI strengths were statistically indistinguishable from the corresponding virgin strengths under the present test conditions. However, the absence of a statistically significant difference does not establish complete strength recovery.

After the 10 J impact, healing produced a significant increase in residual compressive strength for CPUD (*p* = 0.027) and UD (*p* < 0.001), whereas the increase observed for CW was not statistically significant (*p* = 0.145). Nevertheless, the healed 10 J groups remained significantly different from their respective virgin conditions for all three configurations (CPUD: *p* = 0.023; UD: *p* < 0.001; CW: *p* = 0.009), indicating that the compressive strengths after healing had not fully recovered to the corresponding virgin levels. Thus, the statistical comparisons support partial recovery after 10 J, while the healing-efficiency estimates provide a measure of the fraction of the impact-induced strength loss recovered.

In thermally activated self-healing composite systems, recovery is primarily associated with matrix- and interface-dominated damage, where the mobilized healing phase can flow into and bridge cracks and delaminated regions [11,12]. Its effectiveness is therefore limited by the amount and mobility of the healing phase, crack opening, and damage severity, while irreversible damage such as fiber fracture cannot be fully restored by this mechanism [9,12].

The observed recovery of residual compressive strength can also be compared with results reported for other self-healing composite systems. Mao et al. [38] investigated CFRP laminates containing core–shell nanofibers and reported healing efficiencies of 66.3%, 43.5%, and 8.83% at increasing impact energies, respectively. Bhatia et al. [39] demonstrated that thermal reconsolidation of thermoplastic CFRP laminates restored the compressive strength to approximately 100% of the undamaged value after 20 and 30 J impacts, whereas recovery after a 40 J impact was limited to 85%. More recently, Nandi et al. [40] reported recovery of approximately 90% and 62% of the original compression-after-impact strength for two vitrimer-based CFRP systems. Comparable energy-dependent behavior was also observed by Cohades and Michaud [12] for E-glass/epoxy-PCL composites, with CAI healing efficiencies decreasing from 95.9% at 8.5 J to 73.0% at 17 J and 22.4% at 34 J following thermal treatment at 150 °C for 30 min. Although direct quantitative comparisons are limited by differences in material systems, laminate geometries, impact energies, healing procedures, and definitions of recovery efficiency, these studies support the general observation that post-impact strength recovery depends strongly on damage severity and the mechanisms available for its repair. The present results are broadly consistent with these findings, showing reduced healing efficiency at the higher impact energy for the investigated laminate configurations.

A limitation of the present study is that unimpacted control specimens were not subjected to the same thermal treatment (150 °C for 30 min). Therefore, the possible contribution of additional curing or other thermally induced changes in material properties cannot be separated from the recovery associated with the self-healing mechanism. Consequently, the reported healing efficiencies should be interpreted as the apparent recovery of compressive strength following thermal treatment rather than as a direct measure of the contribution of the self-healing mechanism alone.

## 4. Conclusions

The low-velocity impact response, internal damage characteristics, residual compressive performance, and post-impact healing behavior of three self-healing CFRP laminate configurations differing in thickness, reinforcement architecture, stacking sequence, and constituent material system were investigated at impact energies of 5 and 10 J. The main conclusions are as follows:

The 1.45 mm UD laminate showed the lowest maximum contact force and largest out-of-plane displacement, indicating a deformation-dominated response, whereas the 3.30 mm UD laminate exhibited the highest force and smallest displacement. The 2.66 mm woven laminate showed an intermediate response. Thus, the same nominal impact energy was accommodated through different combinations of contact loading and global deformation depending on laminate configuration.

Increasing the impact energy from 5 to 10 J was accompanied by more pronounced changes in the XPCI response in all investigated configurations, while the apparent spatial patterns differed between laminates. Because the XPCI assessment was qualitative, these observations should not be interpreted as quantitative differences in damage area or severity. In the UD laminates, impact-induced changes were not necessarily evident from the surface appearance but were visible in the XPCI images, whereas the woven laminate exhibited a localized change in surface appearance around the impact region. The results demonstrate that global force–time characteristics and surface inspection alone are insufficient to characterize the internal damage state.

The effect of impact energy on residual compressive strength was strongly configuration-dependent. The 1.45 mm UD laminate retained 89% and 82% of its virgin strength after 5 and 10 J, respectively. The 3.30 mm UD laminate retained approximately 98% after 5 J but only 64% after 10 J, representing the largest relative strength loss. The woven laminate retained 88% and 72%, respectively. After 10 J, the woven laminate had the highest absolute residual strength (213.98 MPa), whereas the 1.45 mm UD laminate had the highest normalized strength retention (82%). These results confirm that absolute residual strength and normalized strength retention provide complementary measures of post-impact performance. In particular, among the investigated configurations, a stiffer impact response was not consistently associated with greater residual strength retention.

Thermal healing partially restored the impact-induced loss of CAI strength, with recovery extent depending on both impact energy and laminate configuration. After 5 J, all three laminates exhibited approximately 98–99% strength retention following thermal treatment. The 1.45 mm UD laminate achieved a healing efficiency of 86%, whereas healing efficiency was not considered meaningful for the 3.30 mm UD and 2.66 mm woven laminates because of their negligible initial strength loss. After 10 J, retained strength increased to 91%, 79%, and 81% for the 1.45 mm UD, 3.30 mm UD, and woven laminates, with healing efficiencies of 49%, 42%, and 31%, respectively. Thus, higher impact severity generally reduced the recoverable fraction of the impact-induced strength loss. Therefore, post-healing strength retention and healing efficiency should be considered complementary metrics for impact-induced strength recovery.

Overall, the results show that impact stiffness, internal damage, residual strength, and healing response provide complementary measures of self-healing CFRP performance. However, because thickness, reinforcement architecture, stacking sequence, and constituent material system were not varied independently, the findings should be regarded as a comparative assessment of the investigated configurations. Future work should decouple these factors, expand the impact-energy range, and introduce quantitative XPCI damage characterization using validated segmentation procedures and appropriate configuration-specific reference images to better relate internal damage characteristics to residual performance and recovery.

## Figures and Tables

**Table 1:** Material systems, reinforcement types, and thicknesses of the investigated CFRP laminates.

Laminate ID	Material System	Reinforcement	Thickness, mm
UD-1.45	T700-UD200-36RW-300	UD	1.45 ± 0.02
UD-3.30	T700-UD200-36RW-300	UD	3.30 ± 0.05
W-2.66	T300-TW200-42RW-1250	woven	2.66 ± 0.05

**Table 2:** Laminate properties of the samples.

Parameter	UD-1.45	UD-3.30	W-2.66
No. of plies	8	16	14
*E*_1_, MPa	133,400	133,400	66,600
*E*_2_, MPa	6000	6000	66,600
*G*_12_, MPa	5000	5000	5980
*ν*_12_	0.28	0.28	0.04
*A*_11_, kN/mm	79.75	182.72	137.86
*A*_22_, kN/mm	79.75	182.72	137.86
*A*_66_, kN/mm	28.17	64.58	55.49
*D*_11_, Nm	22.60	272.0	75.57
*D*_22_, Nm	7.68	92.51	75.57
*D*_66_, Nm	3.51	42.26	38.43

**Table 3:** Experimental groups, impact energies, healing conditions, and numbers of specimens.

Specimen Set	Laminate ID	Impact Energy, J	Healing
CPUDV	UD-1.45	0	No
CPUD1	UD-1.45	5	No
CPUD1h	UD-1.45	5	Yes
CPUD2	UD-1.45	10	No
CPUD2h	UD-1.45	10	Yes
UDV	UD-3.30	0	No
UD1	UD-3.30	5	No
UD1h	UD-3.30	5	Yes
UD2	UD-3.30	10	No
UD2h	UD-3.30	10	Yes
CWV	W-2.66	0	No
CW1	W-2.66	5	No
CW1h	W-2.66	5	Yes
CW2	W-2.66	10	No
CW2h	W-2.66	10	Yes

**Table 4:** Statistical parameters of the low-velocity impact response of the investigated CFRP laminates.

Specimen	Maximum Force, F			Maximum Energy During Impact, E			Maximum Displacement, d_h_		
	Mean, N	Std, N	CV, %	Mean, J	Std, J	CV, %	Mean, mm	Std, mm	CV, %
CPUD1	2133	138	6.5	5.29	0.003	0.1	6.25	0.067	1.1
CPUD2	3053	41	1.4	10.35	0.015	0.1	8.04	0.079	1.0
UD1	2968	36	1.2	5.29	0.03	0.6	3.44	0.075	2.2
UD2	4084	294	7.2	10.23	0.08	0.8	4.66	0.390	8.4
CW1	2525	110	4.4	5.32	0.03	0.5	3.90	0.149	3.8
CW2	3142	102	3.2	10.31	0.012	0.1	5.51	0.197	3.6

Std—standard deviation; CV—coefficient of variation.

**Table 5:** Average residual compressive strength and corresponding statistical parameters obtained from repeated CAI tests of CFRP laminates (*n* = 5).

Specimen	Residual Compressive Strength, MPa	Std, MPa	CV, %	*R_σ_*, %	*K*, %
CPUDV	193.63	5.00	2.6	100	0
CPUD1	173.11	11.23	6.5	89	11
CPUD2	159.41	5.09	3.2	82	18
UDV	206.49	10.02	4.9	100	0
UD1	201.68	8.68	4.3	98	2
UD2	131.53	4.93	3.7	64	36
CWV	295.86	21.07	7.1	100	0
CW1	259.76	2.99	1.2	88	12
CW2	213.98	16.35	7.6	72	28

Std—standard deviation; CV—coefficient of variation; *R_σ_*—retained compressive strength; *K*—damage index.

**Table 6:** Post-healing CAI strength, strength retention, healing efficiency, and corresponding statistical parameters of the investigated CFRP laminates (*n* = 5).

Specimen	CAI After Healing, MPa	Std After Healing, MPa	CV, %	*R_σ_*, %	*η_h_*, %
CPUD1h	190.74	5.59	2.9	99	86
CPUD2h	176.27	7.64	4.3	91	49
UD1h	201.65	6.37	3.2	98	n.a.
UD2h	163.11	6.04	3.7	79	42
CW1h	294.19	22.95	7.8	99	n.a
CW2h	238.96	12.67	5.3	81	31

Std—standard deviation; CV—coefficient of variation; *R_σ_*—strength retention after healing; *η_h_*—healing efficiency.

## Data Availability

The data that support the findings of this study are available from the corresponding author upon reasonable request.
